# Basidiomycete pigments as sustainable food colorants and stabilizers: from fungal biology to industrial potential

**DOI:** 10.3389/fmicb.2025.1725536

**Published:** 2025-12-11

**Authors:** Samantha C. Karunarathna, Saowaluck Tibpromma, Wenhua Lu, Hansika Perera, Entaj Tarafder, Baggya Sharmali Karunarathna, Dong-qing Dai, Jaturong Kumla, Chathurika Karunanayake, Kalani Kanchana Hapuarachchi, Nakarin Suwannarach

**Affiliations:** 1 Center for Yunnan Plateau Biological Resources Protection and Utilization & Yunnan International Joint Laboratory of Fungal Sustainable Utilization in South and Southeast Asia, College of Biology and Food Engineering, Qujing Normal University, Qujing, China; 2 Center of Excellence in Microbial Diversity and Sustainable Utilization, Chiang Mai University, Chiang Mai, Thailand; 3 Department of Biology, Faculty of Science, Chiang Mai University, Chiang Mai, Thailand; 4 Zest Lanka International (Private) Limited, Polonnaruwa, Sri Lanka; 5 Department of Chemistry, Faculty of Science, Eastern University Sri Lanka, Chenkalady, Sri Lanka; 6 Office of Research Administration, Chiang Mai University, Chiang Mai, Thailand; 7 Department of Botany, Faculty of Natural Sciences, The Open University of Sri Lanka, Nugegoda, Sri Lanka; 8 College of Biodiversity Conservation, Southwest Forestry University, Kunming, China

**Keywords:** basidiomycete pigments, circular bioeconomy, mushroom-derived food colorants, natural food stabilizers, sustainable food additives

## Abstract

Pigmented Basidiomycete fungi are emerging as multifunctional and environmentally friendly substitutes to man-made food coloring. In addition to their bright colors, pigments from these fungi, including melanin, pulvini acids, carotenoids, and phenoxazines, also exhibit potent antioxidant, anti-microbial, and even potential therapeutic effects. Fungal pigments offer greater stability under processing conditions compared to those of plant origin and can be cost-effectively produced by biotechnological culture, particularly agro-waste-based fermentation systems. This review provides an overview of the chemical diversity, biosynthesis, and extraction of pigments from food and non-food Basidiomycetes such as the genera *Cantharellus*, *Pycnoporus*, *Boletus*, *Pleurotus*, and others. Particular emphasis is on their applications in the food and nutraceutical industries, challenges in scaling up and regulatory aspects, and future prospects of fungal biotechnology as a renewably available source of natural pigments.

## 1 Introduction

Natural pigments derived from microorganisms, including bacteria and fungi, have garnered increasing attention due to their biocompatibility, bioactivity, and potential for sustainable production (Delna et al., 2025). Among these, fungal pigments, especially those from Basidiomycetes, are often more promising than bacterial pigments due to their higher stability, broader color range, and easier scalability through fermentation. The global food market is witnessing unprecedented shifts toward clean-label, plant-based, and sustainable foods, of which natural colorants form a rapidly growing market. This shift is driven by growing consumer aversion toward artificial food colorants, such as tartrazine, sunset yellow, and erythrosine, that have been linked with health problems ranging from child behavioral abnormalities, allergies, and potential carcinogenicity (European Food Safety Authority, 2021; de Oliveira et al., 2024). Thus, regulatory agencies like the FDA and EFSA are increasingly certifying natural alternatives (European Food Safety Authority, 2025; FDA, 2025). The scale of additive use is substantial; while the US Food and Drug Administration (FDA) lists over 3,000 food additives, industry estimates put 8,000 to 10,000 individual chemicals in the American food system when including GRAS-classified and self-confirmed ingredients (Neltner et al., 2011; Leasca, 2024).

This can be observed in the global pigment market, worth around 9.7 million metric tons in 2024 and expected to grow from a worth of USD 26.6 billion in 2025 to more than USD 41 billion in 2033. While a lot of volume previously headed to the paint and textile industries, pigment use in food items has risen substantially due to the need for safer, microbe- or plant-derived colorants (Venil et al., 2014; Chatragadda and Dufossé, 2021; Pillai et al., 2024; Pigments market, 2024; IndexBox, 2025). However, most plant colorants are still handicapped by problems like seasonality of supply, geographically restricted availability, and process condition instability. While bacterial pigments offer a microbial alternative, they often face limitations in color diversity, yield, and scalability compared to fungal alternatives. In this context, Basidiomycete fungal pigments are a perfect, multi-purpose, and ecologically friendly choice. Basidiomycete pigments are more stable to heat, light, and pH than the majority of plant and Ascomycete pigments. Apart from that, they possess a rich variety of classes that vary from melanin, pulvini acid derivatives, styrylpyrone derivatives, terphenylquinones, phenoxazines, to carotenoids, which possess intrinsic bioactive activity such as antioxidant, antimicrobial, and anti-inflammatory activities (Velíšek and Cejpek, 2011; Stadler and Hoffmeister, 2015; Lin and Xu, 2020; Negi, 2025).

While pigmentogenic bacteria and Ascomycetes have been widely studied (Nigam and Luke, 2016), Basidiomycete pigments offer distinct advantages of greater environmental stability and economic scalability through fermentation. Basidiomycetes can be cultivated year-round on agro-industrial wastes, such as sugarcane bagasse, corn husk, and coffee grounds, through solid-state or submerged fermentation, making scalable, sustainable production and a circular bioeconomy feasible (Zhang et al., 2021). Fungi like *Pleurotus ostreatus*, *Cantharellus cibarius*, *Pycnoporus cinnabarinus*, and *Laetiporus sulphureus* are of particular interest due to their pigment composition and GRAS potentiality, as compounds like cinnabaric acid and laetiporic acid show intense coloration as well as potent bioactivity (Velíšek and Cejpek, 2011; Mukherjee et al., 2017; Singh et al., 2025). The relatively underexploited value of pigment-producing Basidiomycetes, combined with their inherent functionality and scalable production, positions them at the forefront of future natural food coloring. This review provides a comprehensive analysis of the chemical diversity, biosynthesis, and extraction of pigments from a range of edible and inedible Basidiomycetes. Special emphasis is given to applications in the food and nutraceutical industries, the issues encountered at the scale-up and regulatory approval stages, and the future prospects of fungal biotechnology as a sustainable and functional natural pigment source.

## 2 Major pigment classes from basidiomycetes: chemistry and biosynthesis

### 2.1 Pulvinic acids and derivatives (yellow to orange)

**Figure 1:**
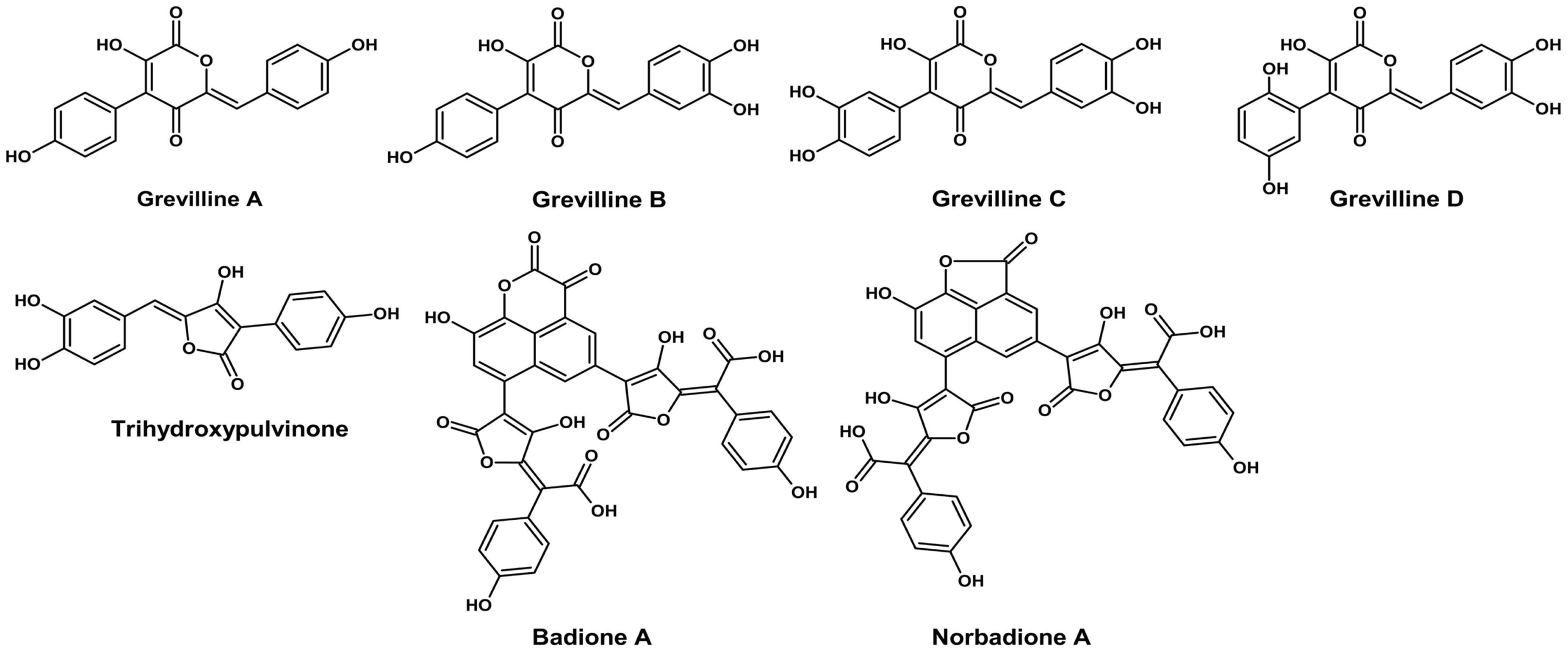
Chemical structures of pulvinic acids and derivatives (ChemDraw v22, 2022).

Pulvinic acid derivatives are yellow-orange pigments predominantly found in Basidiomycetes. For instance*, Suillus grevillei* produces grevillins and trihydroxy pulvinic acid, while *Boletus badius* yields badione A and norbadione A, which create bright yellow to golden brown hues (Figure 1). These compounds exhibit strong metal-chelating and antioxidant properties, making them highly suitable for natural food applications, such as snack coatings, cheese substitutes, and spice powders. Relative to the pigments of Ascomycetes, the distinctive structure and dual purpose of the pulvinic acids reflect the sophisticated biosynthetic abilities of the Basidiomycetes. According to Lin and Xu (2023), these pigments are included within the broad classes of fungal pigments and represent a characteristic group of the Basidiomycota.

### 2.2 Polyketide pigments biosynthesis in basidiomycota

**Figure 2:**
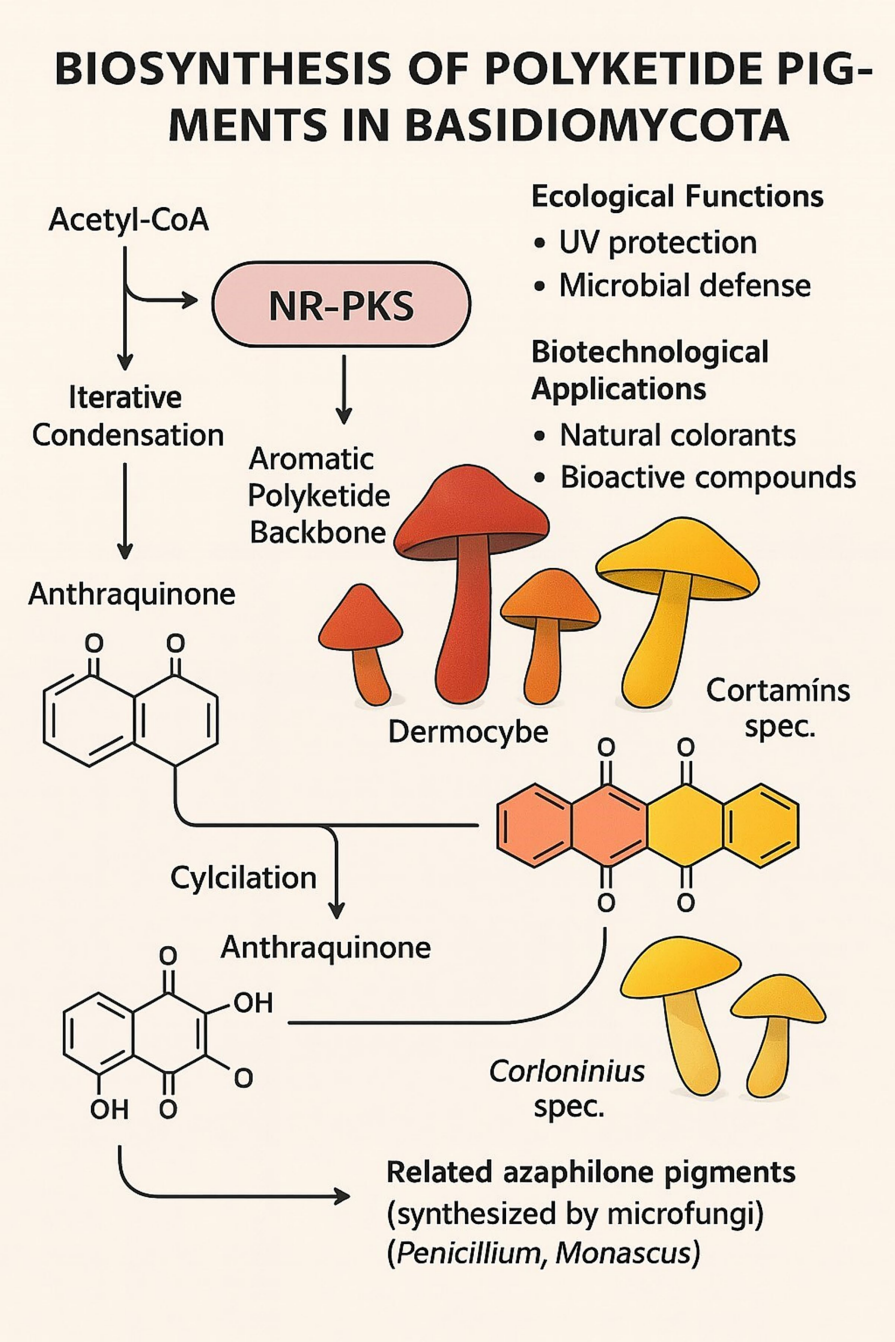
Biosynthesis of polyketide pigments in basidiomycota.

Basidiomycota polyketide pigments are primarily biosynthesized by the action of type I non-reducing polyketide synthases (NR-PKSs), which drive the synthesis of complex aromatic compounds (Figure 2). This section details the major classes of these pigments and the unique biosynthetic machinery that produces them.

#### 2.2.1 Anthraquinones

Anthraquinones are very typical polyketide pigments responsible for the deep red, orange, and yellow fruiting body colors in species such as *Dermocybe* and *Cortinarius* (Gill et al., 1992; Haritha and dos Santos, 2021). The pigments are biosynthesized through iterative condensation of acetate units derived from acetyl-CoA using NR-PKS enzymes as catalysts. The resultant polyketide backbone is then subjected to typical cyclization and oxidation reactions, which in most instances are catalyzed by tailoring enzymes, to yield the characteristic anthraquinone structure. Besides their role in coloration, pigments play ecological roles as UV screens and predators- and microbes-defense compounds and hold valuable biotechnological potential as natural dyes and bioactive molecules (Haritha and dos Santos, 2021).

#### 2.2.2 Styrylpyrones

A significant class of polyketide-derived pigments in Basidiomycota is the styrylpyrones, notably produced by therapeutically relevant genera such as *Phellinus* and *Inonotus* (Lee and Yun, 2011). Their biosynthesis proceeds through a type III polyketide synthase (PKS) that catalyzes the condensation of malonyl-CoA with a cinnamoyl-CoA starter unit, followed by cyclization (Löhr et al., 2023). These yellow pigments are polyphenolic compounds with demonstrated antioxidant and anti-inflammatory activities. Structurally, they exhibit convergence with plant flavonoids (Lee and Yun, 2011). While often overlooked in favor of high-molecular-weight polysaccharides like *β*-glucans, styrylpyrones represent a fascinating group of natural products with potential for functional food formulation and synergistic applications with other fungal polyketides (Olennikov and Gornostai, 2023).

#### 2.2.3 Biosynthetic machinery of basidiomycete polyketides

The core enzymatic machinery for polyketide pigment synthesis in Basidiomycota consists of non-reducing PKSs (NR-PKSs). These mega synthases construct aromatic polyketide backbones with limited reduction, which facilitates the formation of quinone and aromatic structures found in pigments like anthraquinones and styryl pyrones.

A key feature of many basidiomycete NR-PKSs, such as *Cortinarius odorifer* CoPKS1/4, is their distinct domain architecture (KS-AT-PT-ACP-TE; Löhr et al., 2022). This architecture includes a product template (PT) domain that guides specific cyclization and a thioesterase (TE) domain that catalyzes the release of the full-length polyketide chain. This iterative catalytic mode generates remarkable structural diversity, which is not easily achieved by plant or bacterial PKS systems. These enzymes tend to catalyze the formation of hepta- and octaketides, yielding precursors of the ubiquitous pigments emodin and rufoolivacin.

Evolutionarily, the NR-PKSs of basidiomycetes belong to a divergent clade from their ascomycete counterparts. This is highlighted by comparison with azaphilone pigments, the homologous polyketides, which are produced by microfungi such as *Penicillium* and *Monascus* (Keller et al., 2015). Similar biosynthetic pathways notwithstanding, the hallmark difference lies in the fact that many ascomycete anthraquinone synthases rely on external thioesterases, whereas many basidiomycete enzymes possess an integrated TE domain. This enzymic divergence emphasizes a divergence in fungal secondary metabolism that occurs during evolution, even as both groups converge to the formation of intricate aromatic pigments.

### 2.3 Carotenoids (golden yellow)

**Figure 3:**
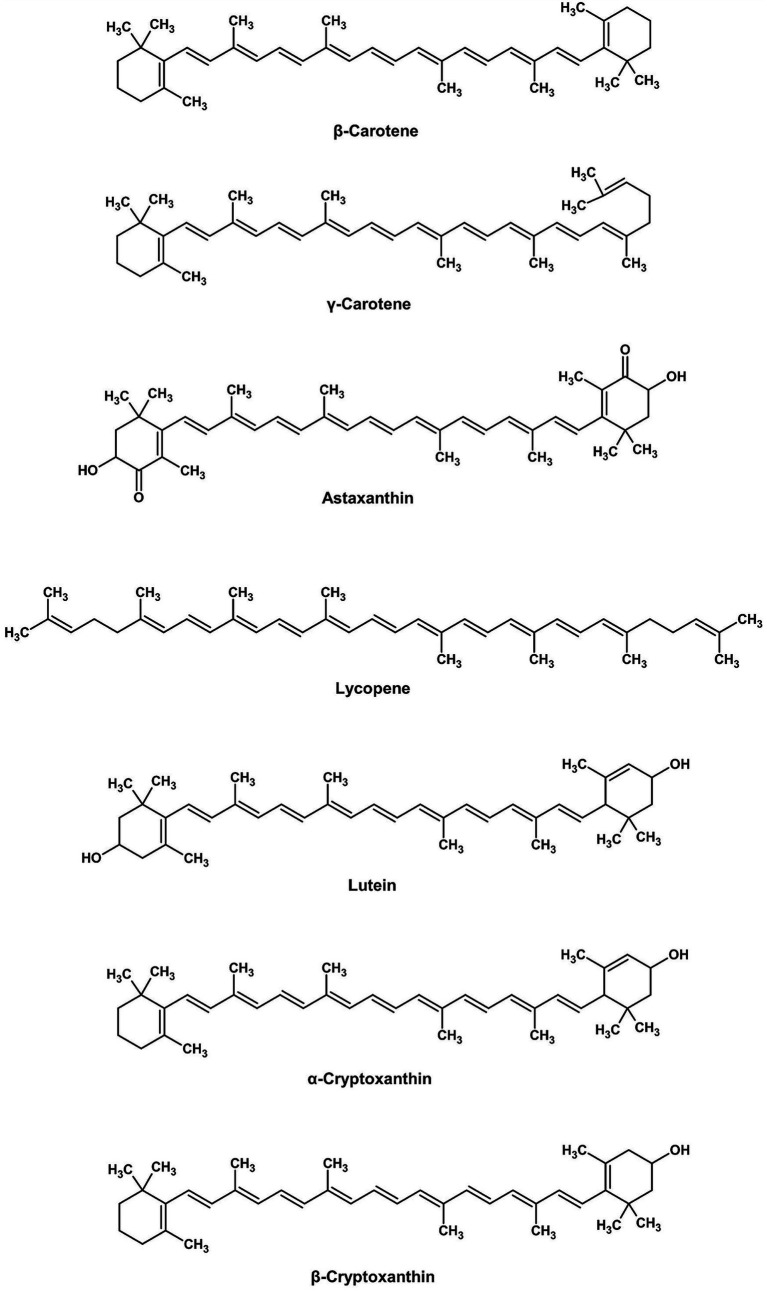
Chemical structures of carotenoids (ChemDraw, v22, 2022).

*Cantharellus cibarius* produces *β*-carotene and lycopene (Figure 3), lipid-soluble pigments that have excellent antioxidant activity, enhancing fat-containing foods such as butter substitutes and sauces. These pigments have good thermal and light stability over plant origins. Besides direct coloring, fungal metabolites interact synergistically with other pigments, enhancing the total effect. Moreover, even non-pigment fungal compounds can significantly improve the stability of industrial colorants, thereby extending their shelf life and performance.

#### 2.3.1 Carotenoid biosynthesis in basidiomycota

**Figure 4:**
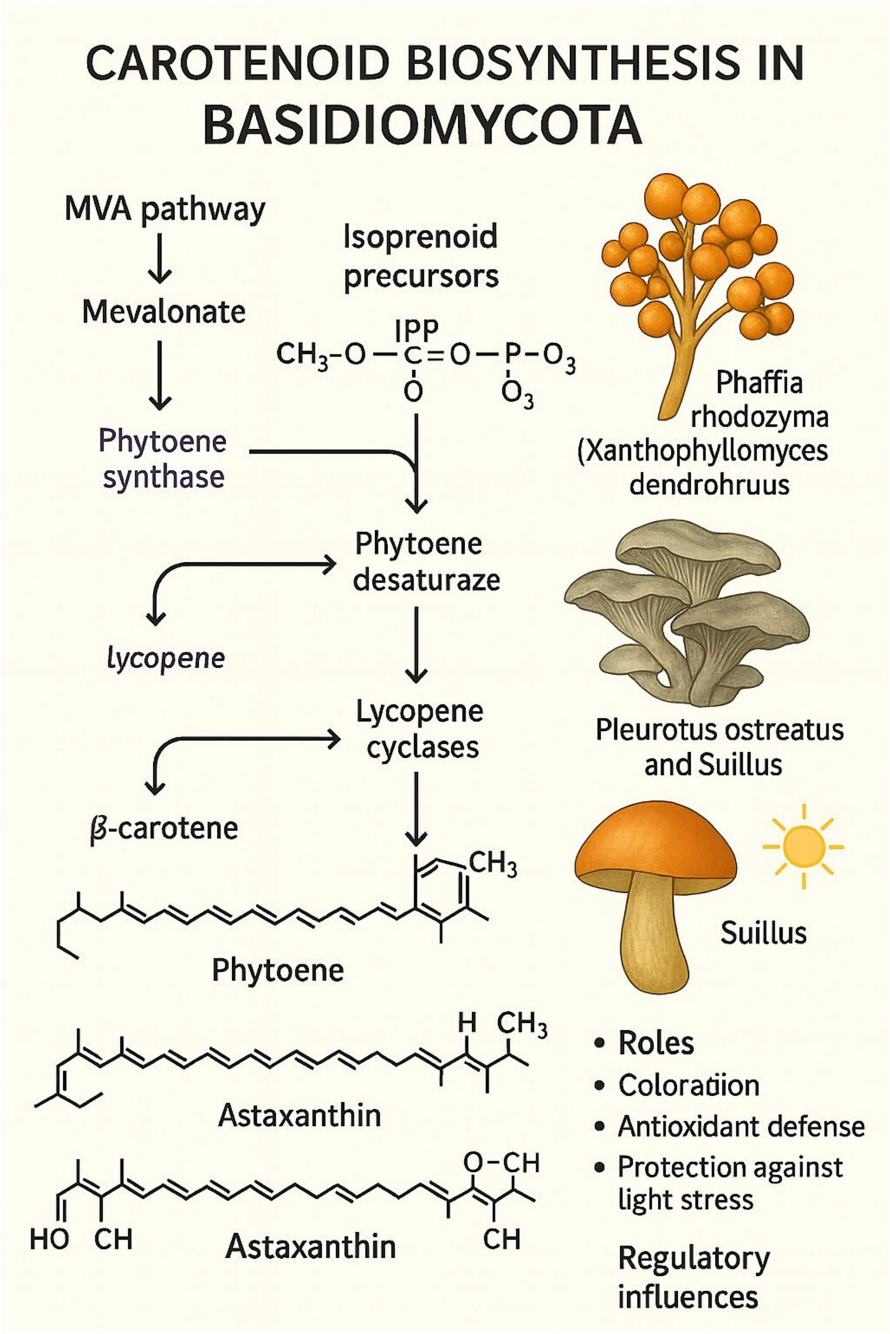
Biosynthesis of carotenoids in basidiomycota.

Carotenoids in Basidiomycota are terpenoid pigments synthesized from the mevalonate (MVA) pathway, which produces the essential isoprenoid precursors isopentenyl pyrophosphate (IPP) and dimethylallyl pyrophosphate (DMAPP). The subsequent biosynthetic pathway proceeds through a defined series of enzymatic reactions to form colored carotenoids (Figure 4). The pathway begins with the condensation of IPP and DMAPP to form the C20 backbone, geranylgeranyl pyrophosphate (GGPP). Two molecules of GGPP are then condensed tail-to-tail by the enzyme phytoene synthase to produce the first colorless carotenoid, phytoene. This compound is subsequently desaturated by phytoene desaturase, a series of reactions that introduce double bonds to form the red pigment lycopene. Finally, lycopene cyclase enzymes catalyze the formation of cyclic end groups, converting lycopene into colored carotenoids such as *γ*-carotene and *β*-carotene.

Apart from contributing yellow, orange, or red pigmentation to basidiomycete fruiting bodies, these pigments are critical for antioxidant protection and light stress protection (Avalos and Limón, 2015; Haritha and dos Santos, 2021). One of the most extensively researched carotenoid-producing Basidiomycota is *Xanthophyllomyces dendrorhous* (teleomorph of *Phaffia rhodozyma*), a producer of astaxanthin that exhibits strong antioxidant activity and has extensive applications in aquaculture, cosmetics, and functional food products (Martínez-Moya et al., 2021).

Species such as *Pleurotus ostreatus* and those in the genus *Suillus* sequester β-carotene, a pigment that regulates their antioxidative stress protection and coloration (Haritha and dos Santos, 2021). The biosynthetic regulation of these compounds is typically linked to environmental stimuli such as light and oxidative stress, but their regulatory mechanisms are less characterized than in microfungal models. While Basidiomycota-specific carotenoid pathways tend to preserve their core enzymology, they exhibit characteristic pigment compositions and ecological and physiological roles. In contrast with the model microfungal targets of industrial pigment production, carotenoid-producing basidiomycetes represent under-exploited but promising sources of biotechnological pigments.

### 2.4 Phenoxazines (orange to red)

**Figure 5:**
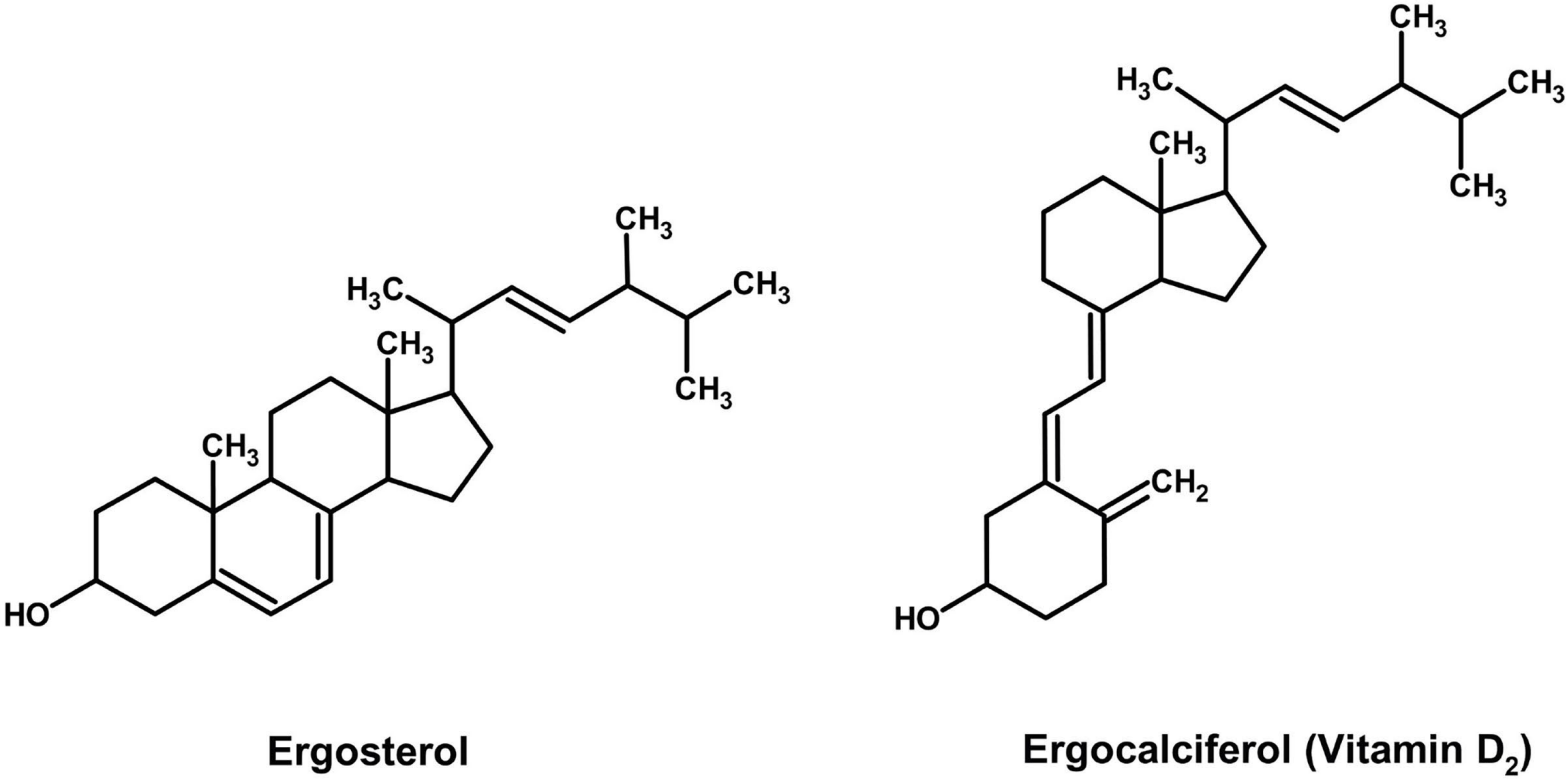
Chemical structure of cinnabarinic acid (ChemDraw, v22, 2022).

Phenoxazines are tricyclic heterocyclic compounds whose core structure consists of a fused ring system containing oxygen and nitrogen atoms. In fungi, they are typically synthesized through the enzymatic oxidation and dimerization of precursor compounds. A well-known biosynthetic route involves the laccase-mediated coupling of ortho-aminophenols, resulting in the formation of the distinctive phenoxazine ring. A prominent example is *Pycnoporus cinnabarinus*, which produces cinnabarinic acid (Figure 5), an orange-red pigment with applications in bakery toppings, fruit glazes, and confectionery. This type of pigment, however, has yet to be fully exploited since wild-type strains produce it in low yield.

### 2.5 Melanins (black to brown) and UV-responsive pigments in basidiomycetes

**Figure 6:**
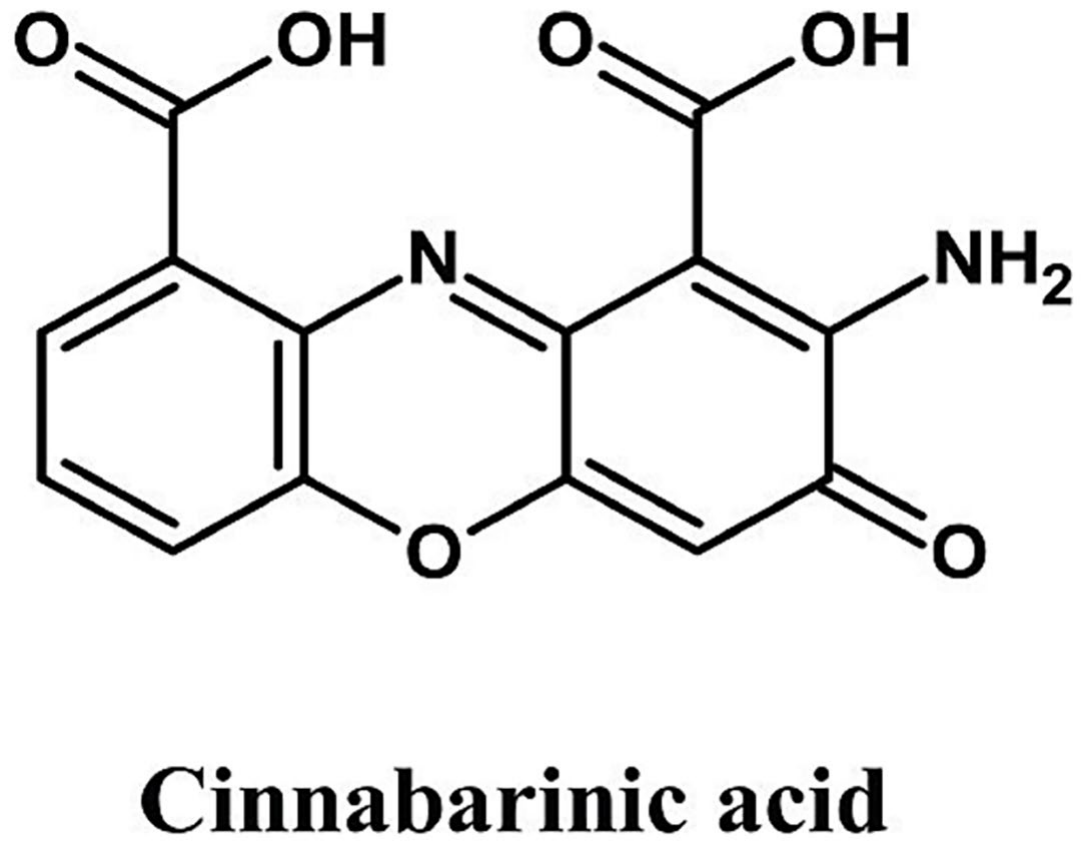
Chemical structure of melanins (ChemDraw, v22, 2022).

Fungal melanins (Figure 6) are one of nature’s most functional biomaterials with exceptional antioxidant and radioprotective activities through efficient free radical scavenging and electromagnetic wave absorption (Mattoon et al., 2021).

The superior function of the melanin is attributed to the stable phenolic radicals within its complex polymeric network, while multiple carboxyl groups enable exceptional heavy metal chelation, broadening its functional applications from pigmentation to medical radiation shielding and environmental remediation (Mattoon et al., 2021).

In Basidiomycetes, *P. ostreatus* (black, yellow, and pink strains) and *Auricularia auricula-judae* produce melanins of different colors depending on the eumelanin to pheomelanin ratio. The melanins also possess potential applications as natural substitutes for caramel colorants used in soft drinks and sauces, with the added benefit of protection against UV light and extended shelf life. The enzymatic melanin biosynthetic pathway in such fungi shares fundamental similarities with that of human tyrosinase, tyrosine hydroxylase, and DOPA oxidase activities (Hornyak, 2018). There are, however, substrate binding pocket variations between fungal and human tyrosinases and the absence of cross-kingdom efficacy of thiamidol-type inhibitors, which suggests Basidiomycete melanins are more resistant to degradation by human skin enzymes and potentially more stable food colorants.

Besides UV protection, Basidiomycete melanins have strong radioprotective activity. Accordingly, in murine models, *A. auricula-judae* melanin provided 80% survival after lethal 9 Gy irradiation, better than synthetic melanin by dual Compton scattering and free radical scavenging mechanisms (Revskaya et al., 2012). All of this makes them promising bioactive materials for radiotherapy adjuvants, food for space missions, and nuclear event emergency rations. Functional variation is important in melanins: the darker-gray *P. ostreatus* exhibits greater anticancer activity toward HT-29 colon carcinoma cells, but its antioxidant activity is surpassed by that of yellow *P. cornucopiae*, illustrating how biological activity and functional trade-offs are influenced by pigment class (Kim et al., 2009).

Some Basidiomycetes also produce UV-reactive pigments in photoprotection. Melanized fungi, such as *Cryomyces antarcticus* (Pacelli et al., 2020) and dark-pigmented forms of *Pleurotus*, produce UV-protecting melanins that could be applied as natural alternatives to chemical UV protectants in functional foods and biodegradable packaging materials. Carotenoid- and mycosporine-producing mushrooms, such as *Pycnoporus*, and high-altitude yeasts (Libkind et al., 2009) exhibit enhanced carotenoid and mycosporine production under UV stress. These traits could be utilized in outdoor or controlled-environment systems for production. Control of the light regime is a useful method for optimizing pigment production and activity. *Shiraia bambusicola*, for example, grown under alternating 24-h light/dark cycles, showed an improvement of 73% in hypocrellin pigment production (Sun et al., 2018). Parallel approaches would stimulate phenoxazine accumulation in *Pycnoporus* or carotenoid levels in *Cantharellus*. Observe that light-dependent pigment responses are taxon-specific; in *Monascus*, red light causes pigment production and blue light inhibits it, while Basidiomycete pigments such as cinnabarinic acid show contrary responses. This emphasizes the necessity for taxon-specific light regulation in controlled fermentation and industrial biotechnological processes. Combined, these attributes demonstrate the multifaceted potential of Basidiomycete melanins and UV-inducible pigments as sustainable, bioactive dyes with potential applications in food conservation, health enhancement, and the development of green materials.

#### 2.5.1 Melanin biosynthesis pathways in basidiomycota

Eumelanin-type pigments in mushrooms such as *Agaricus bisporus* and *Russula nigricans* are produced via the gamma-glutaminyl-4-hydroxybenzene (GHB) pathway. The biosynthetic pathway is identical to melanin biosynthesis in animal tissues. Cata-lytic oxidation of GHB by tyrosinase results in the formation of eumelanin pigments, with nitrogen being relocated from the glutaminyl unit, but not to the glutamyl moiety, to the pigment molecule. Such new nitrogen supplements have nutritional significance when melanins are employed as food functionality or pigment, bestowing nitrogenous value to foods and enhancing pigmentation. Despite having been documented since 1980, the mechanism remains unexplained (Eisenman and Casadevall, 2012; Upadhyay and Xu, 2018).

**Figure 7:**
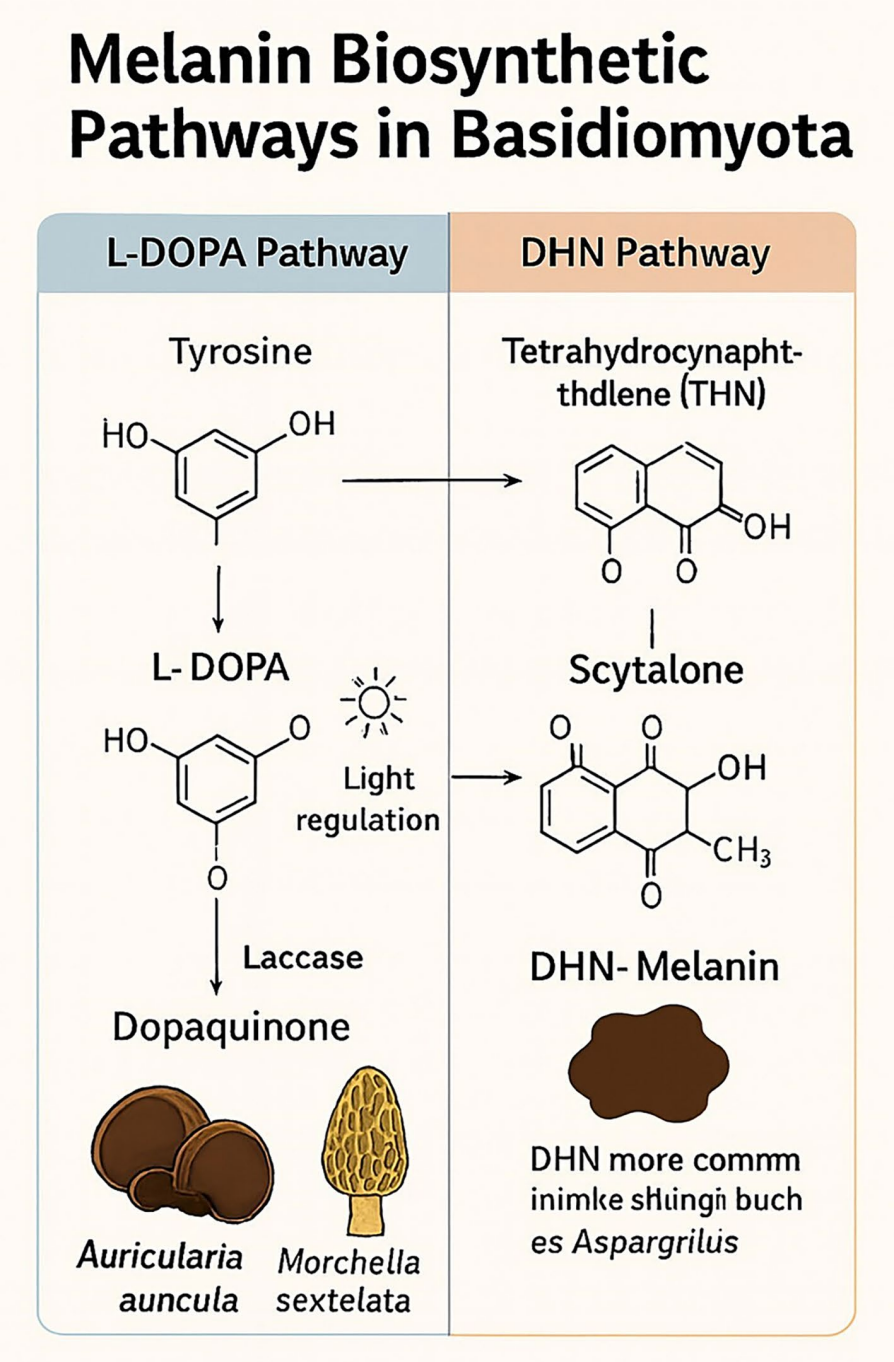
Melanin biosynthetic pathway.

Fungal melanin pigments are black, high-molecular-weight polymers whose primary protective functions are against environmental stresses, including ultraviolet (UV) light, oxidative stress, and desiccation. Melanin biosynthesis (Figure 7) in Basidiomycota typically occurs via two biosynthetic pathways: the L-DOPA (3,4-dihydroxyphenylalanine) pathway and the DHN (1,8-dihydroxynaphthalene) pathway (Eisenman and Casadevall, 2012; Upadhyay and Xu, 2018). The pathway of L-DOPA starts with the enzymatic oxidation of L-tyrosine to L-DOPA, which is oxidized to dopaquinone. Spontaneous and enzymatic conversion results in DOPA-melanin, a eumelanin-type melanin.

The process is enzymatically catalyzed by tyrosinase (EC 1.14.18.1), which hydroxylates tyrosine and oxidizes L-DOPA, and by laccase (EC 1.10.3.2), which oxidizes phenolic intermediates to polymerize (Bell and Wheeler, 1986; Eisenman and Casadevall, 2012). Various Basidiomycota contain this pathway, e.g., *Auricularia auricula-judae* causes L-DOPA-induced precipitation of melanin in fruiting bodies (Upadhyay and Xu, 2018). Blue light significantly stimulates the genes of melanin laccase and tyrosinase in *Morchella sextelata* (a morel fungus with a Basidiomycota-like habit), linking melanogenesis to photomorphogenesis in mushroom-forming fungi (Wang F, et al., 2021). It is important to note that while *Morchella* produces a mushroom-like fruiting body, it is taxonomically a member of the Ascomycota, not the Basidiomycota. This indicates that the L-DOPA melanin pathway, while predominant in Basidiomycota, can also be present and functionally significant in certain Ascomycetes.

The common DHN pathway of Ascomycota, including *Aspergillus* and *Fusarium*, starts with a pentaketide skeleton formed by the action of polyketide synthases (PKSs). The dehydration skeleton is reduced to yield precursors such as scytalone and Verme lone, which are then oxidized and polymerized to form DHN-melanin (Bell and Wheeler, 1986). Less common in Basidiomycota, some members of the orders Tremellales and Auriculariales within this group contain hybrid or partial DHN pathways (Eisenman and Casadevall, 2012; Upadhyay and Xu, 2018). Compared to Ascomycota, well-investigated DHN-melanin pathways, genetics, and enzymes (e.g., *Fusarium graminearum* and *Aspergillus nidulans*). Basidiomycota are highly reliant on the L-DOPA pathway; however, genomic data suggest that metabolic plasticity can be achieved, and a few basidiomycetes possess PKS-like genes involved in DHN-melanin biosynthesis, a case of convergent evolution and biochemical divergence (Gadd, 1982; Eisenman and Casadevall, 2012).

*Cryptococcus neoformans* pheomelanin is a fluorescent hydrophobic pigment granule derived from homogentisic acid (HGA) by HGA oxidation that increases fungal pathogenicity. The fungus also produces neuromelanin-like pigments in the brain using catecholamines, such as norepinephrine and dopamine, from the host and increases its pathogenicity.

With the exception of most of the Ascomycota, most of them produce DHN-melanin by the pentaketide pathway, but some basidiomycetous yeasts (e.g., *Phaeococcomyces*) also produce DHN-melanin, but very infrequently in Basidiomycota. The majority of the basidiomycetes are non-Ascomycota and utilize the hydroxynaphthalene pathway typical of Ascomycota. One of the most important distinctions between these phyla, therefore, is that Ascomycota biosynthesize predominantly DHN-melanin, while Basidiomycota biosynthesize predominantly tyrosinase-dependent eumelanin (GHB → eumelanin) or pheomelanin (HGA → pheomelanin). Biotransformation of two pigments, *Cryptococcus neoformans*, differs from other fungi in that it biotransforms host neurotransmitters into pyomelanin and neuromelanin-like pigments.

On the contrary, Basidiomycete fungal melanins are more resistant to degradation compared to the synthetic ones. Naturally produced fungal melanins are robust molecules that are less susceptible to photo-degradation, heat denaturation, and pH instability, and thus are superior natural food colorants and functional ingredients for food additives. Synthetic melanins are most commonly associated with unreproducible polymer molecular structures and low antioxidant activities, and thus are limited to food uses. Basidiomycete melanins, however, confer deep pigmentation and exhibit antioxidant, UV-screening, and possibly health-promoting activities, and can be marketed as renewable, multicomponent food additives (Lin and Xu, 2020; Nguyen et al., 2024).

Pigmented Basidiomycetes, being primarily mushroom genera, are also increasingly being revealed, besides filamentous fungi, for antioxidant activity. A comparative examination of six *Pleurotus* species revealed that pigmented species, such as the pink *P. djamor* and *P. ostreatus*, were more effective antioxidants than the white ones, and this was correlated with their phenolic content. Significantly, the high radical-scavenging activity of *P. citrinopileatus* at low phenolic content, suggesting a probable contribution from non-phenolic pigments or polysaccharides. These results confirm the commodity value of mushroom pigments as food bioactive, justifying their consideration for use in cultivation programs for the production of bioactive products (Mansuri et al., 2017).

#### 2.5.2 Biotechnological and pathogenic significance of melanin

Melanin in *C. neoformans* enhances virulence by protecting against host immune responses. Mushroom tyrosinases (e.g., those from *A. bisporus*) are widely utilized in both commercial and research applications. It is important to clarify that other compounds studied in pigmented mushrooms, such as agaritine from *A. bisporus*, are not melanin intermediates. Agaritine is a phenylhydrazine derivative investigated for its potential antiviral effects, but it is also a known toxicant. Some mushroom melanin intermediates (e.g., agaritine) have been studied for potential antiviral (anti-HIV) effects, though they may also inhibit melanin formation. Beyond canonical melanin pathways, oyster mushrooms employ specialized chromoproteins for pigment stabilization. The *P. salmoneostramineus* pink chromoprotein (23.7 kDa) binds a 3H-indol-3-one prosthetic group and interacts with Mg^2+^—suggesting a novel metal-coordinated melanin assembly mechanism (Valdez-Solana et al., 2023). Its constitutive production enables consistent coloration regardless of cultivation lighting.

#### 2.5.3 Melanin-mediated spore longevity in saprotrophic basidiomycetes

Melanin’s protective role is not confined to fruiting bodies but also involves spore resistance. Nguyen (2018) demonstrated that pigmented saprotrophic mushroom-forming fungal basidiospores remain viable for as long as 2.8 years—far longer than unpigmented spores. This is consistent with the UV-absorbing and antioxidant roles of melanin (Section 2.5.1) and reveals an ecological cost–benefit: melanized spores enable long-distance dispersal and delayed germination, while depigmented spores optimize rapid local colonization on good conditions.

At the biotechnological level, it is crucial for inoculum production and strain preservation. Pigmented fungi (such as melanized-spored *Pycnoporus* spp.) can offer intrinsic advantages to industries where culture stability is critical. On the other hand, light-spored fungi (such as *Pleurotus albinus*) require optimized storage protocols. These findings substantiate accounts of melanin’s radioprotective roles in *Auricularia* (Section 2.5.2) and suggest that spore pigmentation could serve as a predictor of stress tolerance upon screening pigment-forming strains.

### 2.6 Isoindole/non-nitrogen heterocyclic pigments (purple to pink)

Recent discoveries have expanded the diversity of Basidiomycete pigments beyond classical categories. Aragezolone from *Auricularia cornea* represents a structurally unique hybrid pigment with pH-responsive color shifts (purple→pink) for smart packaging, antidiabetic potential (anti-*α*-glucosidase activity 3 × > rutin), and light stability but heat sensitivity, suggesting cold-processing applications (Onuma et al., 2025). The flavonoid-like pigments in *Auricularia* spp. (e.g., biochanin A) exemplify how mushroom secondary metabolites blur traditional classification boundaries. While some studies attribute these compounds to fungal biosynthesis via modified PKS pathways (Pukalski and Latowski, 2022), others caution that environmental absorption may contribute to detected levels (Gil-Ramírez et al., 2016).

### 2.7 Phenolic pigments (brown to red)

**Figure 8:**
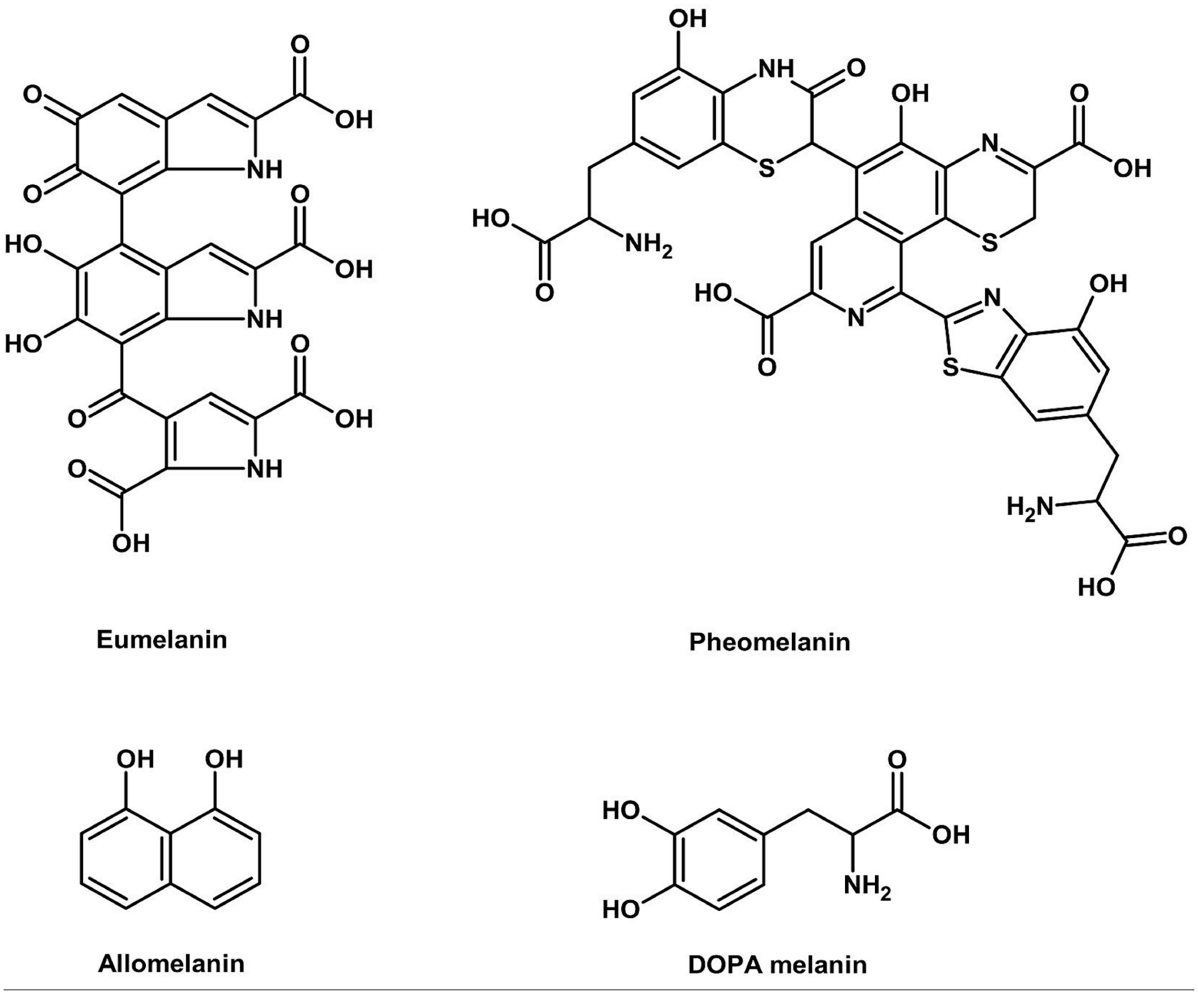
Chemical structures of betalains.

**Figure 9:**
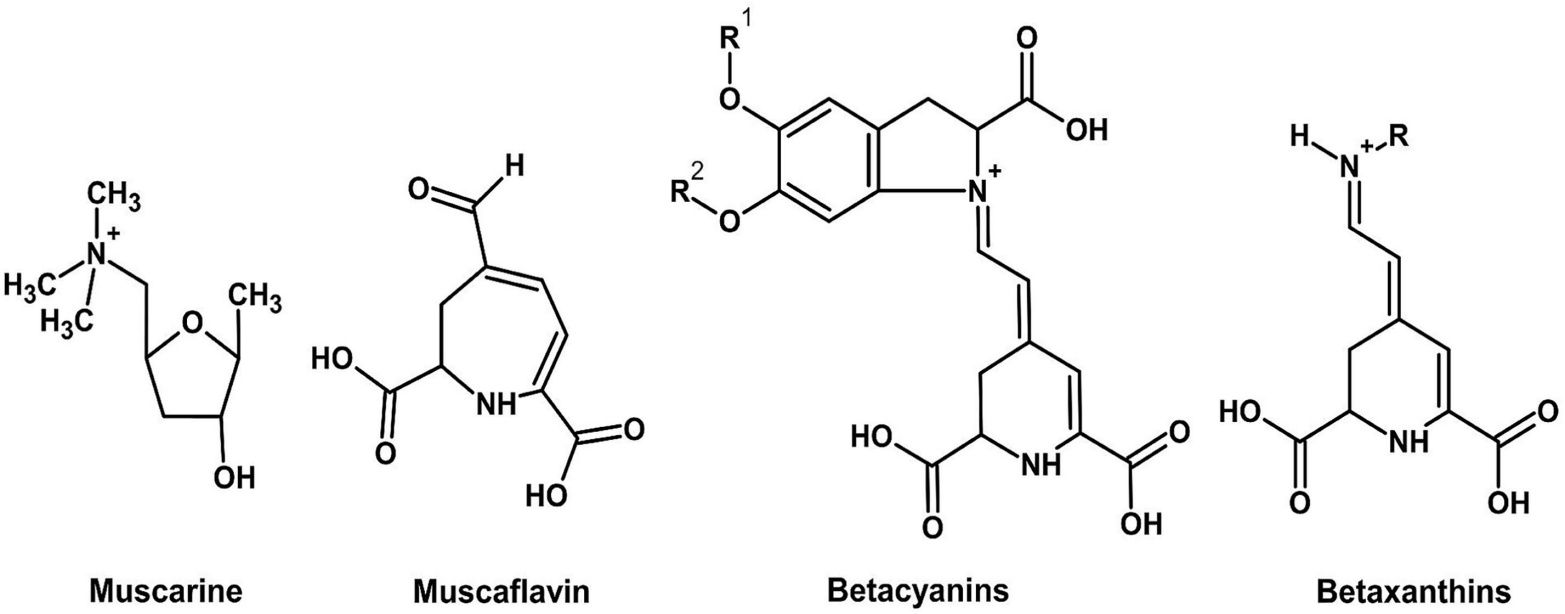
Chemical structures of anthocyanins (ChemDraw, v22, 2022).

*Lentinula edodes* mycelium exudates represent a promising novel source of phenolic-based food color, which provides intense reddish-brown pigments and exceptional stability. A new scientific investigation by Jin (2010) thoroughly examined these exudates, verifying their composition (~5.82% dry weight phenolics) through highly advanced FTICR MS analysis and determining their improved thermal/light stability compared to that of artificial caramel coloring. Key, Jin’s toxicological assays with murine models established a no-observed-adverse-effect level (NOAEL) of 1,000 mg/kg/day for the pigments, indicating that they are safe for food application. Exudate stability—retaining color integrity during high-temperature treatment (e.g., baking, pasteurization)—addresses an important shortcoming of plant phenolics, such as antho-cyanins. Herein, it is also emphasized that production of *L. edodes* pigments through submerged fermentation is scalable, offering a viable pathway to industrial production. Regulatory clearance can be facilitated by the absence of detectable mycotoxins or allergenic proteins in purified fractions. The characteristic chemical structures of betalain and anthocyanin pigment classes are presented in Figures 8, 9, respectively. Table 1 compares the biosynthetic pathways, enzymatic machinery, and taxonomic distribution of major natural pigment classes, highlighting both the unique capabilities of Basidiomycetes and convergent evolution with microfungal producers.

**Table 1:** Biosynthetic origins, key enzymes, and representative fungal taxa involved in the production of major natural pigment types, comparing pigment-producing basidiomycetes with microfungal analogs.

Pigment type	Pathway/origin	Key enzymes	Basidiomycota examples	Microfungi examples	References
Melanin	DHN & L-DOPA	Tyrosinase, Laccase, PKSs	*Auricularia*, *Morchella*; blue-light regulation in *M. sextelata*	*Aspergillus*, *Fusarium* (mostly DHN)	Suthar et al. (2023); Qin and Xia (2024)
Carotenoids	Mevalonate (MVA) pathway	Phytoene synthase, Desaturases	*Xanthophyllomyces*, *Pleurotus*, *Suillus* (β-carotene)	Blakeslea, *Neurospora* (Mucoromycota, Ascomycota)	Mapari et al. (2010); Haritha and dos Santos (2021); Sandmann (2022); Naz et al. (2023)
Polyketide Pigments	Type I PKS polyketides	Non-reducing PKSs	*Dermocybe* (anthraquinones), *Cortinarius*	*Penicillium*, *Monascus* (azaphilones)	Löhr et al. (2022)
Pulvinic Acids	Aromatic amino acid derivatives	Dioxygenases, Oxidases	*Cantharellus*, *Suillus*	Rare in microfungi	Huang et al. (2009); Mudbhari et al. (2024)
Azaphilones	PKS-derived polyketides	PKSs, Oxidoreductases	Rare in Basidiomycota	*Monascus*, *Talaromyces*	Lin and Xu (2020)
Astaxanthin	Modified Carotenoid pathway	β-Carotene ketolase, β-hydroxylase	*Xanthophyllomyces dendrorhous* (*Phaffia rhodozyma*)	Not common	Pavesi et al. (2021)
Betalains	Shikimate pathway	Tyrosinase-like enzymes	Absent	Some ascomycetous yeasts (rare)	Chiang et al. (2009); Tang et al. (2025)

## 3 Applications of basidiomycete pigments

Basidiomycete fungi are a valuable yet underexploited source of natural pigments with high potential for use in the food and aquaculture industries. Pigments, including carotenoids, melanins, pulvinic acid derivatives, and phenolic compounds, exhibit both strong coloration and bio functional activity, such as antioxidant and antimicrobial activity. Unlike synthetic dyes, which are also facing regulatory and consumer resistance, Basidiomycete pigments can be produced on a sustainable large scale through fermentation and exhibit higher stability under process conditions (Mattoon et al., 2021; Zschätzsch et al., 2022).

### 3.1 Pigments for hue replacement

In foods, mushroom pigments are increasingly sought after as natural colorants, being clean-label and sustainability-friendly. *Laetiporus sulphureus*, for example, a yellow-orange polypore, provides pulvinic acid-type pigments that are thermally and acidically stable to a very high extent, making them a possible application in flavored beverages, breads, and fermented foods (Bergmann et al., 2022). In addition, *Pycnoporus cinnabarinus* yields phenoxazinone and cinnabarinic acid pigments from synthesis that exhibit high oxidative and light stability in preliminary food-model systems and offer good alternatives for man-made dyes like Red 40 or Yellow 5 (Gawel, 2024). Basidiomycete pigments are particularly favored as they are more process-stable than their plant counterparts. Unlike the breakdown of anthocyanins from fruits or betalains from beets under alkaline or neutral pH or high temperature, most fungal pigments are not lost in color upon extrusion, pasteurization, or long-term storage (Gawel, 2024). Such stability is desirable in applications in plant-based meat, dairy alternatives, or convenience foods. Regulatory acceptance for hue replacement is also incrementally widening. In Asia, Japan and South Korea have accepted certain pigments of *Schizophyllum commune* and *T. versicolor* in food additive safety frameworks. In the EU, the EFSA is currently reviewing other fungal metabolites for placement in its list of approved colors, i.e., those with low toxicity and well-documented thermal stability (European Food Safety Authority, 2021). This regulatory drive will open up new markets in functional foods, especially in the EU and North America.

### 3.2 Multifunctional additives

Conversely, melanins from food-grade Basidiomycetes, such as *A. auricula-judae* or *L. edodes*, contribute a deep brown to black color and high antioxidant activity, making them suitable for application in functional food coatings, protein bars, and beverage powders (Yin et al., 2022). From the marketing and industry perspective, natural food colors across the globe are likely to reach USD 3.5 billion in 2027, and mushroom pigments are going to hold higher market share because they can act as both color additives and health supplements (Market Data Forecast, 2024). Compatibility with veganism, free-from allergens, and clean-label tag enhances brand reputation, specifically among millennials and Gen Z consumers. Startups and ingredients firms are now looking at branded Basidiomycete pigment extracts, e.g., “MycoHue™” or “FungiTint™,” to develop identity-driven products that offer consumers sustainable sourcing and biotechnology narratives. In addition, fungal fermentation dyes from, e.g., *Hericium erinaceus*, *Marasmius* spp., and *P. ostreatus* enable round-the-year harvests with no seasonality or enormous land and water requirements as with botanical dye plants (Zschätzsch et al., 2022).

### 3.3 Aquaculture feed applications

In aquaculture, fungal pigments, in particular carotenoids, are gaining regulatory and market acceptance as clean-label color intensity enhancers. Astaxanthin, the pigmentation compound for salmonids, is traditionally synthesized from petrochemical precursors or obtained from *Haematococcus pluvialis*. Fungal sources, such as *X. dendrorhous*, offer a 40–60% cost reduction for fermentation-based production compared to artificial approaches (Zantioti et al., 2025). Astaxanthin from *X. dendrorhous* has been accepted by EFSA for salmon and trout diets (European Food Safety Authority, 2021), while *Rhodotorula mucilaginosa* and *Sporidiobolus pararoseus* are found to be beneficial to tilapia, shrimp, and aquatic ornamental fish. The fungi improve the flesh color and oxidative resistance of fish as well as immune responses (Chen et al., 2019; Van Doan et al., 2023). Industry-wise, the aquaculture pigment market exceeds USD 1 billion annually, with more than 75% of the market allocated to salmonids. With increasing consumer resistance to artificial colorants, natural fungal pigments are now being promoted as “clean seafood colorants,” which also provide value through immunomodulation. Furthermore, the positioning can also be utilized by manufacturers to market products as “naturally pigmented,” and this has already been shown to influence consumers’ purchase behavior in North America and Europe (Zantioti et al., 2025). Scale-up is nevertheless limited by fermentation bottlenecks, particularly in optimizing production of lipid-soluble carotenoids. Bioreactor-based solutions using agro-waste substrates and metabolic engineering are being investigated to address this limitation (Li et al., 2022).

### 3.4 Vitamin D₂ biofortification as a value-added co-product

**Figure 10:**
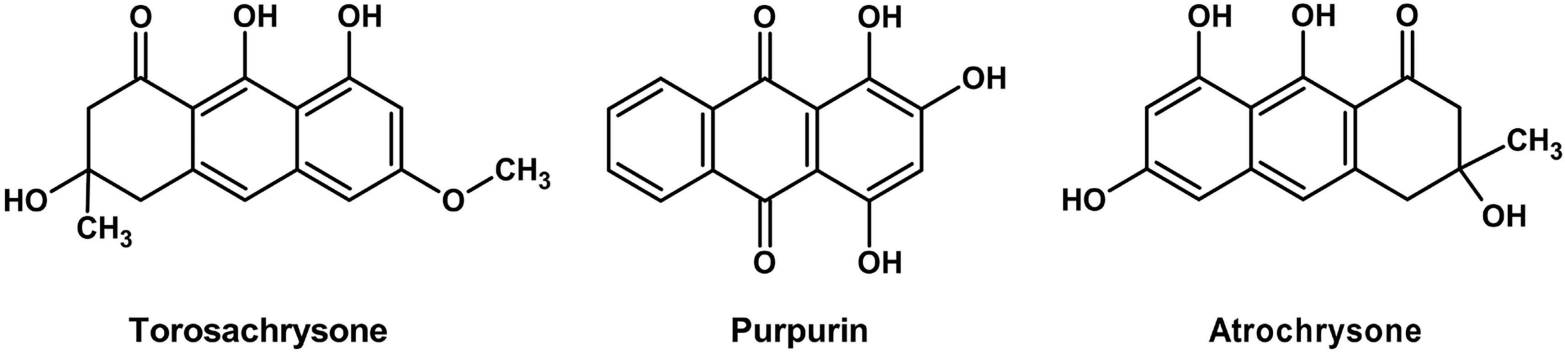
Chemical structures of vitamin D_2_ (ChemDraw, v22, 2022).

The value of pigmented Basidiomycetes extends beyond their chromatic properties to include significant nutraceutical potential. A prime example is ergocalciferol (Vitamin D₂), a secosterol synthesized from the fungal membrane sterol ergosterol upon exposure to UV light. While distinct from pigment pathways, the ability to enhance Vitamin D₂ content through post-harvest UV treatment adds a compelling functional food dimension to mushroom-based colorants. For instance, while *Cantharellus cibarius* is primarily studied for its carotenoid pigments, its fruiting bodies also contain ergocalciferol (Vitamin D₂), which is formed from the photo-conversion of ergosterol upon UV irradiation (Figure 10).

Rangel-Castro et al. (2002) demonstrated that dried chanterelles retain substantial Vitamin D₂ levels (0.12–6.30 μg g^−1^ DW; mean 1.43 μg g^−1^ DW) with great stability even after 2–6 years of storage. The wide variation in concentration—attributable to differential sunlight exposure and not pigmentation (no difference was observed between pigmented and albino strains), highlights the limitations of pooled-sample food tables. Mechanistically, UV-B irradiation is particularly effective at breaking the B-ring of ergosterol to form pre-Vitamin D₂, thereby enhancing the photo-conversion efficiency. This supports the recommendation of controlled UV irradiation during processing to normalize and boost yield. These findings are consistent with research on *A. bisporus*, where post-harvest UV treatment significantly increases Vitamin D₂ content (Koyyalamudi et al., 2009), positioning *C. cibarius* as a species of dual functional relevance for pigment and nutraceutical production via mycelial fermentation. Like carotenoids, UV-inducible vitamin D₂ (ergocalciferol) in *Cantharellus cibarius*, albeit its biosynthesis is from ergosterol precursors, not terpenoid metabolism (Rangel-Castro et al., 2002; Cardwell et al., 2018). Such dual UV responsiveness renders pigmented mushrooms both a nutraceutical and a colorant source.

**Figure 11:**
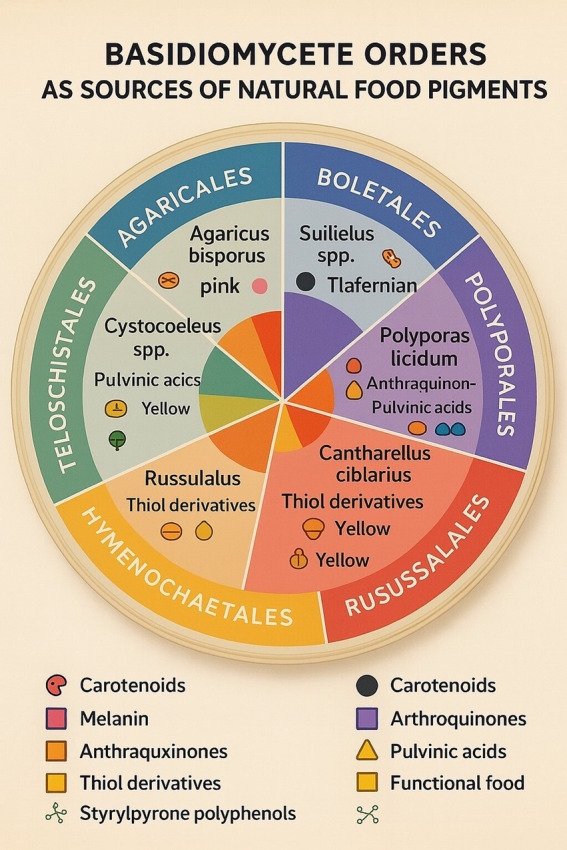
Basidiomycetes as a source of natural food pigments.

Table 2 summarizes the taxonomic distribution of pigment-forming Basidiomycetes with dominant species, their pigment color and type, and known or possible uses in food and commercial products. Figure 11 illustrates the role of Basidiomycetes as a source of natural food pigments. Table 3 summarizes current commercial and emerging mushroom-derived pigments and stabilizers for food applications, highlighting their sources, industrial uses, and market readiness to demonstrate the growing viability of Basidiomycete-based solutions in the food industry.

**Table 2:** The taxonomic distribution of pigment-producing basidiomycetes.

Order	Family	Species	Pigment type	Pigment name	Observed color	Commercial/food relevance	References
Agaricales	Agaricaceae	*Agaricus bisporus*	Carotenoids	Lycopene, β-carotene	Orange to red	Food (antioxidant)	Chattopadhyay et al. (2008)
			Thiol derivative	Ergothioneine (ESH)	White/Brown	Color stabilizer	Dubost et al. (2007)
			Carotenoids (xanthophylls)	Lutein, canthaxanthin, cryptoxanthin	Yellow-orange	Provitamin A, antioxidant, functional food additive	Ribeiro et al. (2011)
			Melanin	Eumelanin	Brown	Food colorant	Mattoon et al. (2021)
			-	-	Brown/Dark	Used in bread, meat products (e.g., sucuk, liver pâté); causes darkening of crumb/surface and impacts sensory scores	Kurt and Gençcelep (2018); Cerón-Guevara et al. (2021)
		*Termitomyces albuminosus*	-	Sulfur-rich melanin	Dark (brown-black)	Food colorant	De Souza et al. (2018)
	Amanitaceae	*Amanita muscaria*	Betalain derivatives	Muscaurins I–VII, Muscaflavin	Orange-red to yellow	Traditional food dye (historical/ethnobotanical)	Li and Oberlies (2005)
		*Amanita* spp.	Betalains	Betalain compounds	Yellow to red	Potential natural colorant	Musso (1979)
	Cortinariaceae	*Cortinarius* spp. (= *Dermocybe* spp.)	Anthraquinones	Various anthraquinones, Torosachrysone	Red, brown, olive green, violet, Orange-Red	Used as natural dyes; valued for stability and light-fastness; potential for food/cosmetics	Gill et al. (1992); Bechtold and Mussak (2009)
	Hygrophoraceae	*Hygrocybe* spp.	Betalains (from tyrosine)	Betacyanins, betaxanthins	Yellow to red	Potential natural colorant, antioxidant	Musso (1979)
	Lyophyllaceae	*Termitomyces* sp.	Not specified	Not specified	Not specified	Potential natural food colorant	Nor-Aleesya et al. (2023)
	Omphalotaceae	*Lentinula edodes*	Thiol derivative	Ergothioneine (ESH)	Tan	Color stabilizer, Retains red color in fish meat under ice storage	Bao et al. (2010)
			Phenolic exudates	-	Reddish-brown	Natural food colorant (stable, safe)	Jin (2010)
	Pleurotaceae	*Pleurotus eryngii*	Thiol derivative	Ergothioneine (ESH)	-	Effective in fish meat preservation; potential additive alternative to ascorbate	Bao et al. (2010)
		*P. cornucopiae*	Thiol derivative	Ergothioneine (ESH)	-	Used for stabilizing color in minced tuna and yellowtail	Bao et al. (2010); Pahila et al. (2017)
		*Pleurotus citrinopileatus*	-	-	Yellow	Potential natural food colorant	Nor-Aleesya et al. (2023)
			Melanin	Allomelanin (DHN pathway)	Bright yellow	High potential as natural yellow dye; heat-stable for processed foods	Medihi et al. (2024)
		*Pleurotus cystidiosus var. formosensis*	Melanin	Melanin	Black	Edible mushroom; pigment studied mainly for characterization, potential in food or biotech	Selvakumar et al. (2008)
		*Pleurotus djamor*	-	-	Pink	Potential natural food colorant	Nor-Aleesya et al. (2023)
			Melanin	Pheomelanin	Pink to salmon	Novel pink colorant for specialty foods	Medihi et al. (2024)
		*P. salmoneostramineus* (*P. djamor*)	Indole-derived chromophore	Indolone (3H-indol-3-one)	Dark reddish-orange (crystals); pink in cap	Potential for functional food or photosensitizer research	Takekuma et al. (1994)
			Chromoprotein	Pink chromoprotein (melanosomal, 3H-indol-3-one-binding)	Pink	Potential in natural colorant and melanin pathway studies	Valdez-Solana et al. (2023)
		*P. ostreatus*	Flavonic pigments	-	-	Potential for natural food pigment development	Babitskaya and Shcherba (1999)
		*Pleurotus* sp. (Black)	Melanin	Eumelanin-rich	Black	Functional pigment (antioxidant, natural dark colorant)	Zhang et al. (2022)
		*Pleurotus* sp. (Yellow)	Melanin	Eumelanin/phaeomelanin mix	Yellow	Natural food colorant (marketed in fresh produce)	
	Physalacriaceae	*Flammulina filiformis*	Melanin (DOPA-derived)	Dopa melanin, Eumelanin (via L-DOPA)	Yellow → brown	highly Relevant to postharvest food quality—contributes to enzymatic browning that affects visual and market value of mushroom	Fu et al. (2022)
		*Flammulina velutipes*	Thiol derivative	Ergothioneine (ESH)	-	Color stabilizer-Prevents discoloration in tuna and beef; effective natural antioxidant	Bao et al. (2010)
				-	Colorless	Prevents lipid oxidation and preserves astaxanthin color in salmon muscle during storage; potential functional food additive	
	Schizophyllaceae	*Schizophyllum commune*	Anthraquinone-like pigment	Purpurin (putative)	Red	bioactive red pigment with potential for natural food colorant or nutraceutical use alongside prebiotic β-glucans	Saetang et al. (2023)
	Strophariaceae	*Stropharia rugosoannulata*	Flavonoid	Quercetin-3-O-glucoside, kaempferol derivatives	White to wine-red	Potential source of natural antioxidant-rich food colorants; temperature-responsive pigment	Shen et al. (2025)
			Isoindole–Isoxazolone derivative	Aragezolone	Purple to pink (pH-dependent: pink at pH 3.0, blue–purple at pH 12.0)	Potential natural food colorant with strong antioxidant, antiglycation, and anti-diabetic activity	Onuma et al. (2025)
Auriculariales	Auriculariaceae	*Auricularia auricula* -judae	Eumelanin	Eumelanin-like polymer	Dark brown–black	Potential as a functional food pigment with antioxidant benefits	Revskaya et al. (2012); Prados-Rosales et al. (2015); Wu et al. (2018)
Boletales	Boletaceae	*Boletus badius* (=*Imleria badia*)	Pulvinic acid	Badione A, Norbadione A	Chocolate brown, golden yellow	Natural pigment, metal complexing in food packaging context	Mohanta (2020)
		*Boletus edulis*	Anthraquino-nes	Atrochrysone derivatives	Red-Brown	Potential colorants	Aumann et al. (1989)
		*B. erythropus*		Pulvinic acid dimers	Yellow, orange-red; blue on oxidation	Edible mushroom with pH-sensitive pigments; potential for use as natural food colorants, especially in low-pH or dry foods	Winner et al. (2024)
		*Chalciporus piperatus*		Sclerocitrin, Chalcitrin, Norbadione A	Lemon yellow to red	Edible bolete with pigment potential	
		*Lactarius indigo*	Terpenoid derivatives	Lactaroviolin, Stearoyldeterrol	Blue	Edible; potential natural blue pigment	Daniewski and Vidari (1999)
		*Suillus bovinus*, *S. collinitus*	Pulvinic acid derivatives	Methyl bovinate, methyl variegatate	Yellow–orange	Chemotaxonomic markers; possible dye use	Besl and Bresinsky (1997)
		*Suillus* spp.	Grevilins	Grevilin A	Yellow to orange	Investigated for dye and colorant use	
		Various Boletaceae Species	Carotenoids (carotenes)	*β*-carotene, α-carotene, *γ*-carotene	Bright yellow to red	Antioxidant, food supplement, natural food pigment	Ribeiro et al. (2011); Durán et al. (2002)
	Gomphidiaceae	*Gomphidius glutinosus*	Pulvinic acids	Gomphidic acid	Yellow	Edible, pigment potential in niche food markets	Knight and Pattenden (1976)
	Melanogasteraceae	*Melanogaster broomeianus*	Polyene	Melanocrocin	Brilliant yellow	Edible truffle-like fungi, pigment bound to phenylalanine	Aulinger et al., 2001
	Sclerodermataceae	*Pisolithus arrhizus*	Pulvinic acid derivatives	Norbadione A	Dark yellow-brown	Dye mushroom (used in artisanal dyeing, potential for food)	Winner et al. (2024)
		*Scleroderma citrinum*	Pulvinic acid derivatives	Norbadione A, Sclerocitrin, Badione A, Xerocomic acid	Yellow-orange	Traditional dye uses, potential in natural food colorants	Winner et al. (2024)
	Suillaceae	*Suillus grevillei*	Pulvinic acids, Grevillins	Grevillins A–D, 3′,4′,4-Trihydroxypulvinone	Yellow-brown	Natural colorant potential (in edible boletes)	Besl and Bresinsky (1997)
Cantharellales	Cantharellaceae	*Cantharellus cibarius*	Vitamin D2	Ergocalciferol	Yellow-Orange (typical chanterelle color)	Significant source of ergocalciferol (vitamin D2) in dried mushrooms; potential for mycelial production of vitamin D2	Rangel-Castro et al. (2002)
			Carotenoids	*β*-Carotene, Lycopene	Golden yellow	Food coloring, flavor, antioxidant	Kozarski et al. (2015); Haxo (1950)
		*Cantharellus cinnabarinus*	Xanthophyll (Carotenoid)	Canthaxanthin	Pink to red-orange	Natural colorant in gourmet foods	Hanson (2008)
				*β*-carotene-type	Yellow, orange, red	Carotenoid-based color via long conjugated double bonds (absorbs 400–500 nm)	Ribeiro et al. (2011)
Cystofilobasidiales	*Xanthophyllomyces*	*Phaffia rhodozyma* (*Xanthophyllomycesdendrorhous)*	Carotenoids	Astaxanthin	Pink	Food industry	Huang et al. (2022)
Hymenochaetales	Hymenochaetaceae	*Inonotus hispidus*	Phenolic (styrylpyrone)	Hispidin, Hispolon	Yellow	Potential for functional foods or natural additives	Ali et al. (1996)
			Melanin	Fungal melanin (IHFM)	Dark brown/black	Water-soluble melanin with antioxidant properties; potential as a functional food additive	Li et al. (2022)
			Melanin	DOPA-melanin	Dark brown/black	Potential for use in health foods and food additives	Hou et al. (2019)
		*Inonotus obliquus*	Melanin	-	Black	Nutraceutical potential	Babitskaya et al. (1998); Lin and Xu (2020)
			Styrylpyrone polyphenols	Hispidin, Inoscavin B	Orange to dark brown	Nutraceutical, possible food-safe antioxidant	Lee and Yun (2011)
		*I. xeranticus*		Styrylpyrone class compounds	Yellow-brown	Bioactive colorant for functional foods	
		*Phellinus igniarius*		Styrylpyrone derivatives	Yellow	Functional food pigment candidate	
		*P. linteus*		Hispidin, Inoscavin A, phelligridins	Yellow to brown	Potential antioxidant/anti-inflammatory food additive	
			Melanin	Melanin	Brown	Used in bread; changes color due to oxidation and caramelisation	Ulziijargal et al. (2013)
Pezizales	Tuberaceae	*Tuber melanosporum*	Melanin	Melanin	Black	Culinary pigment in truffles; natural colorant potential	Harki et al. (1997)
Polyporales	Fomitopsidaceae	*Laetiporus sulphureus*	Non-isoprenoid polyenes	Laetiporic acid A (66), 2-dehydro-3-deoxylaetiporic acid A	Yellow to orange	Edible species, pigments may have potential as food colorants	Davoli et al. (2005)
	Ganodermatacea	*Ganoderma lucidum*	Triterpenoids	Ganoderic acids	Red-Brown	Nutraceuticals	Hasnat et al. (2013)
			Phenolics, Melanin-like	Polyphenols, Melanin	Brown, reddish-dark	Added to noodles, snacks, brandy, wine, and beer; contributes to Maillard reactions, deeper color	Peci’c et al. (2015); Belscak-Cvitanovic et al. (2017); Nguyen et al. (2019); Szydłowska-Tutaj et al. (2021)
	Meripilaceae	*Grifola frondosa*	Thiol derivative	Ergothioneine	Gray	Color stabilizer	Dubost et al. (2007)
	Polyporaceae	*Pycnoporus cinnabarinus*	Phenoxazine	Cinnabaric acid	Cinnabar red	Potential as a natural dye; studied for industrial and biotechnological use	Gripenberg (1958)
				Cinnabarinic acid	Bright red	Potential food application	Lin and Xu (2020)
		*Anthrodia camphorate*	Pigment not specified	-	Darker Brown	Causes visible color change in bread when used as supplement	Ulziijargal et al. (2013)
Pucciniales	Pucciniaceae	*Puccinia distincta*	Carotenoids	β-carotene, γ -carotene	Orange-yellow	Food industry	Davoli et al. (2005)
Russulales	Hericiaceae	*Hericium erinaceus*	Enzymatic browning pigments	Oxidized polyphenols via PPO (tyrosinase, laccase)	Dark brown	PPO inhibition may enhance food stability	Ulziijargal et al. (2013)
			Melanin-like	Possibly Melanin	Light Brown	Used in functional bread; thermal processing causes color shift	
	Russulaceae	*Lactarius* spp.	Azulenes	Azulene (C₁₀H₈)	Blue	Food and beverages, research potential	Dufossé (2016); Caro et al. (2017)

**Table 3:** Commercial and emerging mushroom-derived food colorants and stabilizers for food applications.

Company/brand	Mushroom pigment source	Product/application	Market status	Key notes	References
MycoTechnology	*Pleurotus ostreatus* (melanin-rich extract)	Used in buns, burger patties, and meat analogs to replace caramel color	Pilot-scale (2025 launch)	Partnered with Cargill; heat-stable natural brown pigment alternative; promotes clean-label bakery and protein products	MycoTechnology (2024)
Chinova Bioworks	*Agaricus bisporus* (MycoFiber® melanin)	Natural stabilizer in dairy, juices, and toffees to prevent oxidation/browning	Commercial (NA & EU)	Color-protective agent in white dairy/beverages; 30% shelf-life extension in white wine; under review for GRAS status	Chinova Bioworks (2024)
Four Sigmatic	*Inonotus obliquus* (chaga melanin), *Ganoderma* spp.	Instant coffee mixes, protein powders, and cocoa beverages	Commercial (Health foods)	Pigments add dark brown hue; antioxidant marketing appeal; avoids synthetic coloring	Four Sigmatic (2024)
Biomyc	*Pycnoporus cinnabarinus* (cinnabarinic acid)	Plant-based meat, candies, and snacks	R&D (Pre-commercial)	Bright red/orange hues; shelf-stable; pH- and heat-tolerant; focus on confectionery and meat substitutes	BioMyc (2024)
Better Nature	*Fusarium venenatum* (mycoprotein base, melanin traces)	Tempeh burgers, protein-rich nuggets, sometimes pigmented naturally	Commercial (UK & EU)	Uses natural mycoprotein pigmentation to avoid added caramel coloring	Better Nature (2024)
Small-Scale EU/US Producers	*Lactarius indigo* (azulenes), *Russula* spp.	Gourmet chocolates, blue cheeses, and candies	Artisanal (Niche market)	Pigments extracted from wild mushrooms; used in gourmet items; pigment supply remains seasonal	Chavan et al. (2025)
Colorifix / Mogu / EU-Funded Consortia	*Lentinula edodes*, *Pleurotus* spp. (phenolics & melanins)	Used in edible coatings, biodegradable wrappers, and bakery glazes	Pilot-phase (EU)	Natural color stabilizers for baked goods, and biodegradable coatings with UV protection, color, and antioxidant function	Mogu (2024)
MyForest Foods (formerly Atlast)	*P. ostreatus* (mycelium color and structure)	MyBacon™ (plant-based bacon with natural coloration from mushroom base)	Commercial (US)	Natural browning of strips during frying due to inherent melanin in mycelium layers	MyForest Foods (2024)
AstaReal (DSM)	*Xanthophyllomyces dendrorhous* (astaxanthin)	Approved in seafood, gummies, and nutraceutical candies	Commercial (Global)	Commonly used in salmon but now tested in red-orange functional gummies for kids and sports products	AstaReal (2024)
MycoTechnology + Cargill	Basidiomycete melanins (proprietary strains)	Targeting caramel color replacement in colas, toffees, canned foods	Launch expected 2025	Focused on replacing Class III & IV caramel with non-toxic mushroom-derived brown pigments	Food Navigator (2024)

## 4 Extraction and production strategies

**Figure 12:**
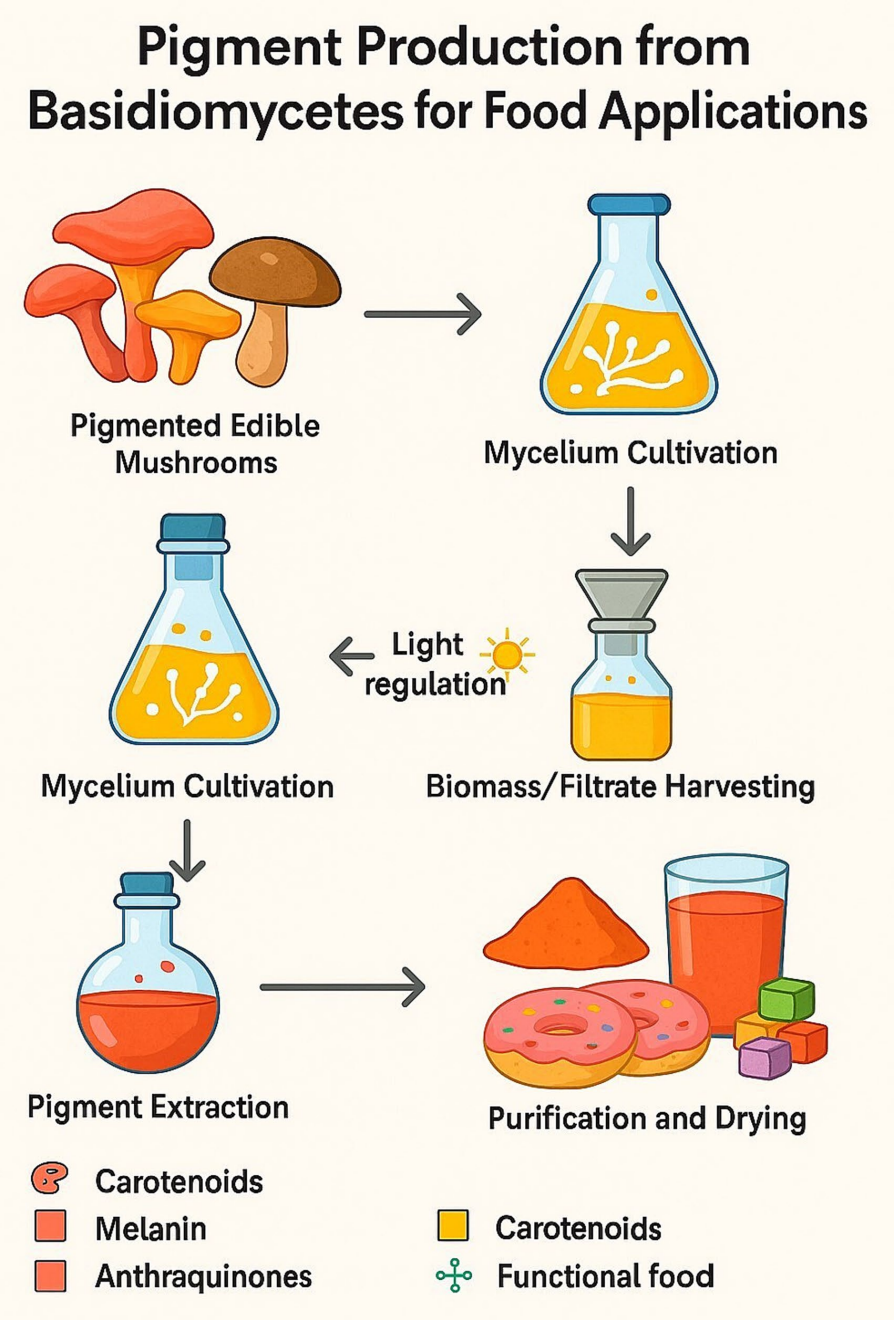
Pigment production process.

Green and sustainable extraction methods are critical to the economic feasibility of Basidiomycete pigments and adhere to green chemistry principles. The general process for producing these pigments, from cultivation to a finished, usable product, is outlined in Figure 12.

Solvent-free, energy-efficient processing with low environmental impact compared to traditional solvent-based processes typically utilized in synthetic dyes is characteristic of such processing methods as supercritical CO₂ extraction. Supercritical CO₂ not only avoids the chemical waste of traditional extraction, but preserves pigment integrity, particularly for thermal- and oxygen-sensitive carotenoids from taxa such as *C. cibarius* (Tauber et al., 2018). Traditional extraction approaches for fungal pigments vary by compound class and fungal species. Alkaline extraction is widely used for melanins from *Pleurotus* species due to the pigment’s insolubility and complex polymeric nature. For more labile pigments such as phenoxazinones and pulvinic acids, ultrasound-assisted and microwave-assisted extraction techniques enable rapid recovery at lower temperatures, minimizing thermal degradation (Kim, 2020). Emerging *in situ* methods, such as Raman spectroscopy, provide non-destructive, real-time monitoring of pigment synthesis and accumulation during fermentation or cultivation, as demonstrated for polyenes like laetiporic acid and pulvinic acids in *Laetiporus* and *Boletus* species (Tauber et al., 2018). This real-time quality control helps optimize harvest timing and extraction efficiency, thereby reducing waste.

The application of controlled oxidative stress, such as hydrogen peroxide treatment, has been shown to significantly enhance pigment production. This treatment often activates stress-response transcription factors (e.g., AP-1, Yap1) that bind to antioxidant response elements (AREs) in the promoters of genes involved in pigment biosynthesis. For instance, pigment yields in *Shiraia bambusicola* increased by 27% following exposure to H₂O₂ (Deng et al., 2016). Such stress-induced upregulation is a promising avenue for boosting the yields of Basidiomycete pigments, such as laetiporic acid or cinnabarinic acid, from *Pycnoporus* and *Boletus*. Temperature also plays a key regulatory role; melanization in *Pleurotus* species is temperature-sensitive, allowing tunable pigment profiles through controlled cultivation parameters (Kim, 2020). Similar to *Monascus purpureus*, where yellow pigment production increases above 45 °C and red pigment biosynthesis is stimulated by NaCl addition, Basidiomycetes show potential for targeted pigment modulation by environmental cues. Cultivation techniques for pigment production include solid-state fermentation using agro-industrial wastes, such as corncobs and banana peels, which offer low-cost and sustainable substrates that also contribute to waste valorization. Submerged fermentation is preferred for standardized and scalable pigment yields, and can be optimized by strain selection, mutation breeding, or metabolic engineering to enhance pigment biosynthesis pathways (Lin and Xu, 2023). Environmental stresses proven to induce pigment production in Ascomycetes, including nitrogen starvation and oxidative stress, also increase Basidiomycete pigment production. Nitrogen starvation, for example, triggers a global stress response that can derepress secondary metabolite pathways, diverting carbon flux toward the synthesis of pigments like cinnabarinic acid in *P. cinnabarinus* (Temp and Eggert, 1999). In contrast to the production of synthetic dyes, which often requires hazardous solvents, high energy inputs, and toxic waste, these biotechnological methods offer cleaner, more environmentally friendly alternatives. Integration of green extraction methods with fermentation processes powered by renewable feedstocks positions Basidiomycete pigments as environmentally responsible solutions for the natural colorant market.

## 5 Synthetic and semi-synthetic approaches

Basidiomycete fungal pigment chemical manufacture is a new multidisciplinary research discipline at the intersection of mycology, organic chemistry, and food science. Natural harvesting from mushroom fruiting structures has been the primary source to date of melanins, carotenoids, and pulvinic acid derivative pigments. This is plagued with inherent limitations of yield, seasonality, and scale-up, nevertheless. Thus, chemical synthesis and semi-synthetic modification of fungal pigments are being researched as viable means to enable standardized, cost-effective, and scalable manufacture suitable for food industry requirements. The rationale of chemical synthesis is to avoid the shortcomings of natural extraction typically plagued by low concentration of the pigment, heterogeneous mixtures, and reliance on agricultural conditions. Chemical pathways yield pure pigments with reproducible properties, allowing for structural modification for enhanced pigment stability, solubility, and color tunability. Moreover, semi-synthetic approaches, naturally occurring fungal precursors chemically converted, provide middle-ground solutions for maximizing pigment characteristics without *de novo* synthesis.

An example is the synthesis of pulvinic acid-type yellow pigments, which were first isolated from organisms such as *Suillus* and *Boletus* species. They are aromatic acids of polyphenolic character with bright yellow-orange color, of great interest as food pigments. Synthetic pathways typically involve oxidative coupling reactions of catechol-type precursors under mild oxidizing conditions, allowing for fine-tuning of the pigment’s color and stability. Atromentin, a key pulvinic acid precursor, was first synthesized by Steglich et al. (1972) via oxidative dimerization of substituted phloroglucinols, establishing the foundation for methods that are still applied in modified form today.

Another class of interest is azulenes, which are aromatic non-benzenoid hydrocarbons containing fused ring systems valued for their unique blue color. These pigments are rare among Basidiomycetes, with *Lactarius indigo* being a particularly prominent natural source. Chemical synthesis of azulenes typically involves the cyclization of substituted cyclopentadiene derivatives with electrophilic aromatic partners, usually under Lewis acid catalysis, to yield these deep blue molecules. While fungal biosynthesis occurs via unique, largely uncharacterized enzymatic pathways, synthetic analogs have been developed that mimic the core structural motifs of fungal azulenes rather than their true biosynthetic routes. These synthetic analogs show promising antioxidant activity and thermal stability, as reported by Wang L, et al. (2021), making them candidates for food-grade colorants.

**Figure 13:**
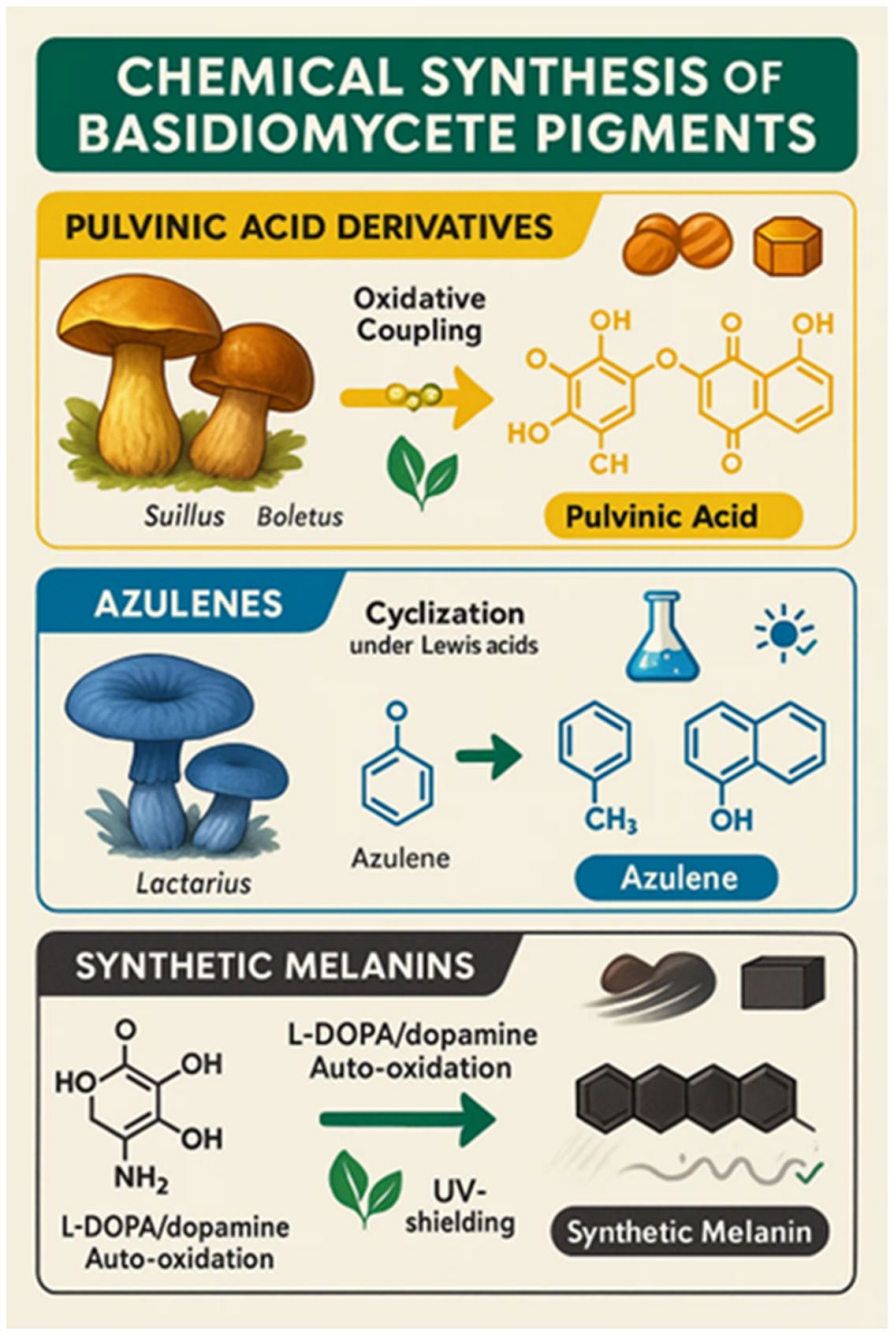
Chemical synthesis of basidiomycete pigments.

Melanin pigments, which are polymeric and heterogeneous in nature, have also been synthetically mimicked through the auto-oxidation of L-DOPA or dopamine under alkaline, aerobic conditions. The primary chemical synthesis routes for recreating major Basidiomycete pigment classes, including pulvinic acid derivatives, azulenes, and synthetic melanins, are summarized in Figure 13.

These synthetic melanins reproduce key physicochemical properties of natural fungal melanins, such as UV protection and radical scavenging, and have therefore been of interest for use in food packaging and edible coating systems. Their deep dark color, along with low solubility, however, has deterred their direct utilization as food colorants. Still, synthetic melanins offer valuable models for studying structure–function relationships and for developing functional materials (Aman Mohammadi et al., 2022).

Despite progress, regulatory clearance of synthetic or semi-synthetic fungal pigment analogs for food applications remains a major hurdle. Compounds must demonstrate non-toxicity, biodegradability, and stability under various food processing conditions to meet safety standards established by agencies such as the FDA and EFSA. Therefore, modern synthetic routes increasingly adopt principles of green chemistry—minimizing the use of toxic solvents, energy, and aggressive reagents—in alignment with sustainability goals.

While the majority of chemical synthesis work is at the pilot or laboratory scale, it represents a critical foundation. Growing consumer demand for natural, consistent food colorants, combined with the inherent limitations of extraction-based supply chains, positions synthetic and semi-synthetic approaches as a promising frontier for the scalable production of Basidiomycete pigments. Further elucidation of fungal biosynthetic gene clusters and metabolite pathways provides insight into stable intermediates and functional motifs that can be tapped or mimicked synthetically, thereby bridging the gap between biology and chemistry in the synthesis of new pigments (Aman Mohammadi et al., 2022).

## 6 Legislation of basidiomycete bio-pigments

As with any food additive, biopigments derived from Basidiomycete fungi will also undergo rigorous regulatory testing prior to acceptance for use in foods. These natural pigments of fungi, i.e., pulvinic acid derivatives, melanins, and mushroom betalain-like pigments such as in *Suillus*, *Cortinarius*, and *Inonotus*, will be examined thoroughly for toxicity, allergenicity, purity, and stability (Poorniammal et al., 2021; Dufossé, 2024). Of greatest relevance to safety assurance is the establishment of extraction and purification methods that effectively eliminate undesirable or potentially toxic fungal metabolites, such as mycotoxins and immunogenic compounds (Kaur and Anand, 2025).

Globally, the regulatory framework for natural colorants is evolving in response to rising consumer demands and technological advances. In the United States, the FDA has historically approved mainly synthetic color additives, such as FD&C Red No. 40 and Yellow No. 5, as well as a few mineral-based color additives, including titanium dioxide (E171) and iron oxides (E172). Still, growing consumer demand for naturally and microbiologically sourced colorants has led to the recent approval and consideration of plant- and microbe-source colorants, such as gardenia blue and butterfly pea flower extract, approved in 2025, as a move toward naturally sourced products gets underway (European Food Safety Authority, 2025; FDA, 2025). In the European Union, food additives, such as colorants, are regulated by Regulation (EC) No. 1333/2008. There are 43 approved colorants permitted for use in foods, and they are predominantly of synthetic or vegetable origin (Nigam and Luke, 2016). Basidiomycete pigments have not yet been sold on an industrial basis as food colorants, but certain compounds, such as mushroom-derived melanins and pulvinic acid analogs, are being actively considered for Generally Recognized As Safe (GRAS) approval or novel food authorization. Their dual mode of action as both pigments and antioxidants optimize their use in the development of clean-label and functional food industries (Poorniammal et al., 2021; Chatterjee and Pandey, 2023). Commercialization is currently being hindered primarily by regulatory constraints, scale-up challenges, and gaps in safety data, but is offset by growing research and development efforts.

It is given the utmost priority to safety testing. Fungal pigments must be tested strictly for mycotoxin content, including citrinin—a non-specific *Monascus* species product—and possible allergenicity, according to Poorniammal et al. (2021). Pigments of *Talaromyces purpureogenus*, for instance, were non-toxic to rodent models of toxicity, while Fusarium species naphthoquinone pigments are of safety concern due to being co-produced alongside toxic metabolites. Basidiomycetes, such as *Pleurotus* and *Pycnoporus*, have been reported to possess acceptable safety profiles; however, toxicity testing, including subacute oral toxicity testing and *Artemia salina* lethality testing, should be standardized to determine their safety for food applications. Regulation acceptance will be a follow-up later based on these data gaps, as with the evolution of the Ascomycete pigment industry (Table 4).

**Table 4:** Regulatory status and industrial potential of basidiomycete-derived pigments across major regions.

Pigment name	Source fungus	Color/class	Function/application	Current regulatory status (USA, EU, Asia)	Key challenges	References
Astaxanthin	*Xanthophyllomyces dendrorhous* (yeast, not Basidiomycete but model case)	Red carotenoid	Aquaculture (salmon feed), antioxidant	USA/EU: Approved for feed use only; not authorized as direct food dye. Asia: Approved in Japan, Korea, China for feed and supplements.	Scaling biotechnological yields; cost competitiveness vs. synthetic; GRAS approval for direct food use pending.	Dufossé (2024)
Variegatic acid / Atromentin	*Suillus grevillei, Boletus* spp.*, Hydnellum* spp.	Yellow to orange, pulvinic acid derivatives	Antioxidant, natural food dye potential	USA/EU/Asia: Not approved; novel food candidate.	Lack of toxicology; limited industrial cultivation; regulatory uncertainty.	Gill (1994)
DHN-Melanin / Allomelanin	*Inonotus hispidus, Phellinus, Auricularia* spp.	Black to brown, melanin pigments	UV protection, cosmetics, antioxidant	USA: GRAS for non-food uses; not food-approved. EU/Asia: Under novel food evaluation.	Complex purification; association with pathogenic fungi; regulatory caution around “melanin” terminology.	Solano (2014); Poorniammal et al. (2021)
Dermocybin / Emodin	*Cortinarius sanguineus, Dermocybe* spp.	Red to orange, anthraquinones	Dye studies; pharmaceutical interest	USA/EU/Asia: Not approved; toxicological risks identified.	Nephrotoxicity and genotoxicity concerns; unsuitable for food applications.	Steglich et al. (1972); Yli-Öyrä et al. (2024)
β-Carotene (fungal)	*P. ostreatus*	Yellow to orange, carotenoid	Provitamin A, antioxidant, food dye	USA/EU: β-carotene approved (synthetic/algal sources), not fungal-derived. Asia: Similar restrictions.	Low yields in mushrooms; algae remain dominant industrial source.	Sandmann (2022); Naz et al. (2023)
Torulene	*Schizophyllum commune*	Yellow to orange, carotenoid	Antioxidant, dye candidate	USA/EU/Asia: Not evaluated; still in early research phase.	Very limited production data; requires toxicological and stability testing.	Naz et al. (2023)
Azaphilones	*Dacrymyces, Tremella, Stereum* spp.	Yellow to red, polyketide-type	Antimicrobial, rare fungal pigments	USA/EU/Asia: Not evaluated.	Chemical instability; pH-dependent reactivity; no regulatory precedent.	Lin and Xu (2023); Dufossé (2024)

## 7 Industrial potential and challenges

Basidiomycete pigments yield excellent industrial benefits due to their natural origin, aligning with the trend of increasing consumer demand for clean-label, vegan, and multifunctional products. They also have nutrition or bioactive properties in most instances, which further make them highly desirable to be utilized in food, cosmetic, and pharmaceutical applications. Secondly, the integration of pigment extraction with established mushroom processing infrastructure offers the potential for both the economic viability and environmental sustainability of mushroom-based biorefineries to be enhanced. Despite these promising conditions, commercialization of Basidiomycete pigments is plagued by a comparatively high number of outstanding issues of critical importance (Table 5).

**Table 5:** List of patented bio pigments and their fungal sources.

Species	Pigment type	Pigment name	Observed color	Commercial/food relevance	References
*Agaricus bisporus*	Carotenoids	β-carotene, γ-carotene	Orange-yellow	Nutritional enhancement, natural food colorant	Vacharathit (2009)
*Pleurotus ostreatus*	Carotenoids	β-carotene, lutein	Yellow-orange	Functional food additive	Vacharathit (2009)
*Xanthophyllomyces dendrorhous* (*Phaffia rhodozyma*)	Carotenoids	Astaxanthin	Pink-red	Aquaculture feed pigment, cosmetics, food coloring	Verdoes et al. (2003)
*Inonotus obliquus*	Melanin	Fungal melanin	Dark brown-black	Antioxidant, antiviral, cosmetics, supplement	Google Patents (1996)

Among the most notable issue is the lack of Generally Recognized as Safe (GRAS) status for the majority of pigments from wild Basidiomycete strains. The local strain yield is usually low and can vary broadly with respect to the genetic background of the strain and culture conditions. The unreliability of yield complicates large-scale production, as well as the reproduction of quality. A critical assessment of these production challenges is provided by the quantitative data on yields and costs for representative pigments (Table 6). The data highlight a stark contrast; while fermented pigments like laetiporic acid and fungal astaxanthin achieve promising titers, others reliant on wild harvests or simple cultivation (e.g., *Cantharellus β*-carotene) exhibit yields that are commercially non-viable, directly impacting their economic competitiveness.

**Table 6:** Quantitative data on production, yield, and cost of selected basidiomycete pigments.

Pigment (source fungus)	Pigment class	Reported Yield (mg/L or mg/kg substrate)	Extraction efficiency/method	Stability and performance	Estimated cost/competitiveness
Cinnabarinic acid *(Pycnoporus cinnabarinus)*	Phenoxazine	~50–150 mg/L (Submerged Fermentation)	Moderate; requires solvent extraction (e.g., ethyl acetate).	Excellent light and pH stability; superior to some synthetic reds.	High. Estimated >10 × cost of Red 40. R&D phase; cost drops with optimized fermentation.
Laetiporic acid A *(Laetiporus sulphureus)*	Polyene	~200–500 mg/L (Submerged Fermentation) (Bergmann et al., 2022)	Ultrasound-assisted extraction can achieve >90% recovery.	High thermal stability; fades under strong light.	Moderate–high. Scalable fermentation demonstrated at pilot scale. Cheaper than saffron but more expensive than curcumin.
Melanin *(Auricularia auricula-judae)*	Eumelanin	~5–8 g/kg (Dry fruiting body) (Wu et al., 2018)	Alkaline extraction (1 M NaOH) efficiency ~70–80%.	Exceptional antioxidant activity (IC₅₀ ≈ 0.1 mg/mL); UV stable.	Moderate. Low-cost substrate (agro-waste) potential. Cost is competitive for high-value functional foods vs. synthetic antioxidants.
β-Carotene (Fungal) *(Cantharellus cibarius)*	Carotenoid	~0.5–2 mg/100 g (Fruiting body) (Haxo, 1950)	Supercritical CO₂ extraction is efficient but capital-intensive.	Good stability; comparable to plant-derived β-carotene.	Not competitive. Yield too low vs. algal production (e.g., *Dunaliella* >400 mg/L).
Astaxanthin (Fungal) *(Xanthophyllomyces dendrorhous)*	Carotenoid	~200–450 mg/L (Optimized Fermentation)	Efficient cell disruption & solvent extraction.	Standard for aquaculture; potent antioxidant.	Moderate. ~40–60% cheaper than synthetic astaxanthin; competitive with *Haematococcus* algal source
Pulvinic acid derivatives *(Suillus* spp., *Boletus* spp.*)*	Pulvinic acid	Data scarce; estimated <100 mg/kg (Fruiting body)	Conventional solvent extraction.	Good pH and heat stability.	High. Reliant on wild harvesting; not commercially viable without cultivation development.

Moreover, fungal pigments are not yet broadly accepted for commercial use, and only some major genera, such as *Pleurotus* and *Cantharellus*, have demonstrated market potential.

Safety is always the first consideration. Basidiomycete pigments, like all fungal pigments, need to be rigorously screened for toxic metabolites. A case in point is the nephrotoxin citrinin, found in *Monascus* species, that can act as an example. *Gyromitra* species contain a toxin gyromitrin, which is why it is required to maintain the pigment safety under control before commercial use. Gene engineering methodologies, such as CRISPR-Cas9 gene editing, offer an encouraging answer to such safety concerns by enabling the targeted deletion of biosynthetic gene clusters for toxin production. Species-specific toxicological testing must be performed, as exemplified by the European Union’s ban on hydroquinone due to safety concerns despite having occurred naturally for centuries. This indicates that safety measures can trump natural status when regulation is in place.

To counteract these issues, some solutions are currently being developed. Genetically modified organisms and adaptive laboratory evolution strategies can be utilized to boost the quantity and range of pigments with improved safety profiles. Mimicking nature’s ecological processes through the co-cultivation of fungi has the potential to increase pigment complexity as well as pigment yield efficiency. Marketing the fungal pigments as “fermented fungal colorants” would also increase consumer acceptance by putting the products in an acceptable and familiar category. The introduction of novel analysis methods, e.g., Raman spectroscopy, promises real-time and non-destructive analysis of pigment biosynthesis during the process of fermentation or co-cultivation, thus accelerating process optimization and strain selection.

Basidiomycete melanins, in particular those from *Auricularia* species, are shown to possess the same functional activities as human-pathogenic fungi, e.g., *Wangiella dermatitidis*, such as radioprotection and metal chelation. These above-mentioned characteristics create new functional food possibilities for humans with high exposure to radiation, e.g., astronauts. The application of *Auricularia auricula* extracts in traditional Chinese medicine to protect against the effects of radiation also attests to their functional viability.

Economically, the global carotenoid industry, currently valued at approximately $1.5 billion and dominated by firms such as DSM, can have its scope of activity broadened to Basidiomycete-derived carotenoids if economically viable methods of obtaining compounds such as β-carotene from the *Cantharellus* mushroom are realized. Melanin-producing mushrooms will also be on hand for niche use in radioprotection for occupational defense, space exploration, and medical radiology.

This future work would include the establishment of dose–response relationships for regulatory approvals, e.g., GRAS status, the impact of food processing on pigment bioactivity, and the characterization of synergistic action with other fungal antioxidants, e.g., ergothioneine in *L. edodes*. While toxin contamination is still a major regulatory hurdle, illustrated by the case of *Monascus citrinin*, CRISPR—Cas9—mediated elimination of the toxin biosynthetic pathway, demonstrated to work for Ascomycetes, holds promise for Basidiomycete pigment producers. Several companies, including start-ups such as Microtechnology, are leading the charge in overcoming these hurdles using innovative fermentation technologies, genetic engineering, and bioprocess optimization to yield commercially important fungal pigments. Despite decades of struggle to optimize yields and regulatory challenges, the prospects of Basidiomycete pigments for industrial applications are being revolutionized by advances in biotechnology and analytical methods.

The commercialization of Basidiomycete pigments is propelled by powerful market trends but is simultaneously constrained by a complex regulatory landscape. On the market front, unprecedented consumer demand for clean-label, natural, and vegan products is driving the food industry away from synthetic dyes like tartrazine and Red 40 (Savastano, 2025). This shift is complemented by the positive perception of fermentation as a natural process, allowing fungal pigments to be marketed as sustainable and innovative (Gomes, 2024). The global natural food colorant market, projected to exceed USD 3.5 billion, represents a significant opportunity (ECHEMI, 2025). Potential applications are diverse, spanning stable colorants for plant-based meats, functional ingredients in nutraceuticals leveraging their inherent bioactivity, and cost-effective alternatives in aquaculture feed, as demonstrated by the success of fungal astaxanthin (Gomes, 2024). However, this promising market potential is tempered by formidable regulatory hurdles. Critically, no Basidiomycete pigment extract currently holds Generally Recognized as Safe (GRAS) status in the US or full novel food authorization in the European Union (Elkhateeb and Daba, 2023). The regulatory path requires extensive and costly toxicological studies including subchronic toxicity and allergenicity assessments, to establish safety. A paramount concern for regulators is the rigorous exclusion of potential co-extracted toxins, such as mycotoxins, from the fungal material (Elkhateeb and Daba, 2023). Furthermore, achieving the batch-to-batch consistency and standardized composition demanded by regulatory agencies for approval remains a significant technical challenge for complex biological extracts. This divergence between robust market pulls and stringent regulatory push defines the current industrial paradigm for Basidiomycete pigments. Navigating this landscape will require the continued integration of biotechnology, robust safety science, and strategic market positioning to fully realize their commercial potential.

## 8 Conclusion

Basidiomycete pigments are in the dynamic area of natural, clean-label food color with bright colors and inherent natural bioactivity and fermentative scalability. As detailed throughout this review, their potential is significant, yet commercialization faces distinct challenges, as summarized in Table 7.

**Table 7:** Summary of the advantages and limitations of basidiomycete pigments.

Aspect	Advantages	Limitations/challenges	References
Source and production	Sustainable production via fermentation on agro-industrial waste. Year-round cultivation, independent of season/climate. Supports a circular bioeconomy.	Low yield from wild strains and fruiting bodies. Strain-dependent variability. Scaling up fermentation is challenging.	Zhang et al. (2021); Lin and Xu (2023); see Section 4 (Extraction and Production)
Functionality	Multifunctional: inherent antioxidant, antimicrobial, and therapeutic activities. Enhanced stability—more resistant to heat, light, and pH than many plant-based colorants.	Some pigments are heat-sensitive (e.g., aragezolone). Bioactivity may be influenced by food processing.	Mattoon et al. (2021); Velíšek and Cejpek (2011); Onuma et al. (2025); see Section 3 (Applications)
Color and application	Broad color spectrum (yellows, oranges, reds, pinks, browns, blacks). Can replace synthetic dyes (e.g., Red 40, caramel color). Dual use as colorant and functional ingredient.	Natural color variation between batches. Limited availability of certain colors (e.g., stable blues). May impart unintended flavors.	Bergmann et al. (2022); Gawel (2024); see Table 2 (Taxonomic Distribution); Section 3.1 (Hue Replacement)
Economic and market	Growing consumer demand for “clean-label” and vegan products. Potential for high-value niche markets (functional foods).	High production costs vs. synthetic dyes. Limited consumer familiarity. Market dominance of established alternatives.	Market Data Forecast (2024); Zschätzsch et al. (2022); see Section 7 (Industrial Potential)
Regulatory and safety	Derived from natural, biological sources. Many source mushrooms have a history of safe consumption. Regulatory approval is progressing.	Lack of GRAS status for most specific pigment extracts. Requires rigorous and costly safety testing. Complex and time-consuming regulatory pathways.	Poorniammal et al. (2021); Dufossé (2024); Kaur and Anand (2025); see Section 6 (Legislation)

However, to unleash the promise into commercial reality, an integrated and focused approach is crucial. Future progress will be driven by potent genetic tools, such as CRISPR-Cas9, for strain improvement, innovative fermentation technologies derived from Ascomycete systems to optimize yield, and nanoencapsulation technologies for enhancing food stability. Concurrently, a concerted effort must be made to obtain good safety data and navigate the regulatory pathways in order to reach the market. Some of the promising candidates such as the red pigments of *Pycnoporus* and functional melanins of *Auricularia* are already making a mark. Finally, the full utilization of these versatile fungal pigments will depend on ongoing cross-disciplinary studies among mycologists, food chemists, and biotechnologists to overcome the final limitations of scale, stability, and application, ultimately leading to a brighter and healthier future for food dyes.
